# Fine-Grained Recognition of Insect Pests from Digital Images: A Survey

**DOI:** 10.1007/s13744-026-01385-8

**Published:** 2026-05-04

**Authors:** Telmo De Cesaro Júnior, Claudio André Lopes de Oliveira, Douglas Lau, Rafael Rieder

**Affiliations:** 1 Federal Institute of Education, Science and Technology Sul-rio-grandense (IFSul), Passo Fundo, RS Brazil; 2 Univ of Passo Fundo (UPF), Post-Graduate Program in Applied Computing, Passo Fundo, RS Brazil; 3 Univ of Passo Fundo (UPF), Post-Graduate Program in Agronomy, Passo Fundo, RS Brazil; 4 Brazilian Agricultural Research Corporation (Embrapa Florestas), Colombo, PR Brazil

**Keywords:** Electronic trap, Integrated pest management, Computer vision, Agricultural entomology

## Abstract

Effective pest management requires accurate and continuous monitoring. This monitoring helps assess population dynamics and guides the development of integrated pest management strategies. Traps used to capture insects are an alternative applied to various crops. However, the identification and manual counting of specimens are time-consuming, require taxonomic knowledge, and depend on the expertise of specialists. Automation could reduce costs, increase accuracy, and enable scalable analyses. Current computer vision and artificial intelligence techniques can quickly and accurately identify objects in digital images. This study presents a systematic review of literature retrieved from multidisciplinary and specialized databases (Scopus, ACM, Web of Science, IET, DBLP, Springer, and ScienceDirect), focusing on the intersections of agriculture, ecology, and computer science. We found 284 studies published between 2020 and 2025. Among them, 57 fulfilled the eligibility criteria, considering applied computing solutions for insect identification and counting using digital images of specimens collected via traps or photographed in situ on plants, in both field and laboratory settings. The findings highlight the use of electronic traps for real-time data collection and improvements in convolutional neural networks, with visual transformers and attention mechanisms for multi-species and fine-grained recognition. They also indicate opportunities to leverage microscopy resources, overcome limitations in the large-scale deployment and integration of electronic trap networks, and integrate real-time monitoring data with forecasting models using weather predictions to promote early warning systems for integrated pest management.

**Supplementary Information:**

The online version contains supplementary material available at https://doi.org/10.1007/s13744-026-01385-8.

## Introduction

Modern agriculture faces increasing challenges in crop protection, as agricultural pests pose a significant threat to food security and global productivity (Yonbawi et al. 2023; Hechen et al. 2024). Traditionally, pest monitoring has relied on laborious and subjective techniques, such as manual plant inspection and insect counting in traps. These methods are not only demanding in terms of skilled labor but are also prone to human error, which complicates large-scale implementation (Khairunniza-Bejo et al. 2024). The requirement for specialized taxonomic expertise to distinguish between morphologically similar species or immature life stages further exacerbates these operational constraints (Yonbawi et al. 2023).

To overcome these barriers, advances in artificial intelligence (AI), particularly deep learning (DL), have driven major progress in agricultural pest monitoring (Rustia et al. 2023). Research conducted in several countries, including China, Brazil, Italy, Taiwan, Portugal, Malaysia, Croatia, and Spain, shows a global effort to develop automated pest identification and counting systems (De Cesaro Júnior and Rieder 2020; Li et al. 2021). These systems are being designed for a wide range of crops. Examples include corn, wheat, rice, cocoa, cotton, citrus, mango, tomatoes, soybeans, apples, pears, strawberries, asparagus, sugar beets, witloof chicory, and various greenhouse crops.

DL techniques have been central to these advances. Researchers have used a wide range of intelligent models and custom configurations for object detection, as well as for pest classification and counting in digital images. Several solutions have been proposed in this context. One example is the use of electronic traps (e-traps) with integrated cameras and edge computing devices that rely on light, color, or pheromone attractants. These systems enable continuous real-time monitoring and reduce the need for human intervention (Wang et al. 2022; Rustia et al. 2021; Freitas et al. 2022). Another approach is laboratory scanning and identification, which allows the capture of high-resolution images in controlled environments and supports the use of more complex models (Ibrahim et al. 2023; Kalfas et al. 2023; De Cesaro et al. 2022).

Overall, these technological innovations primarily provide strong technical support for integrated pest management (IPM). These systems provide accurate real-time detection and pest population counting, enabling informed decision-making about the timing and intensity of interventions. That also contributes to reduced pesticide use and promotes more sustainable and less ecologically impactful agricultural practices (Rustia et al. 2023).

This review presents a survey that examines recent advances in automated insect monitoring, focusing on how computer vision (CV) and AI techniques can address the limitations of manual identification. This survey reviewed the technological advances reported in 57 articles, analyzing the state of the art in identifying and counting agricultural pests in challenging scenarios. It highlights the DL methods employed, model performance in generalist, multi-species, and fine-grained classifications, the maturity of e-traps, and the main knowledge gaps.

This survey presents an updated and expanded review of the work previously published by De Cesaro Júnior and Rieder (2020), which focused on developments from 2015 to 2019. The necessity for a new bibliographic survey covering the 2020–2025 period is driven by rapid technological advancements over the past 6 years that have fundamentally transformed the paradigm of computer vision (CV) in agriculture. Notable innovations include convolutional neural network (CNN) architectures from the YOLO family and Vision Transformers (ViT). In conjunction with the widespread adoption of high-performance edge computing platforms such as NVIDIA Jetson, Raspberry Pi, and ESP32, these advances have enabled the transition from theoretical models to autonomous embedded solutions. This study seeks to elucidate these recent developments and to provide guidelines for monitoring systems aligned with the principles of Agriculture 4.0.

**Fig. 1:**
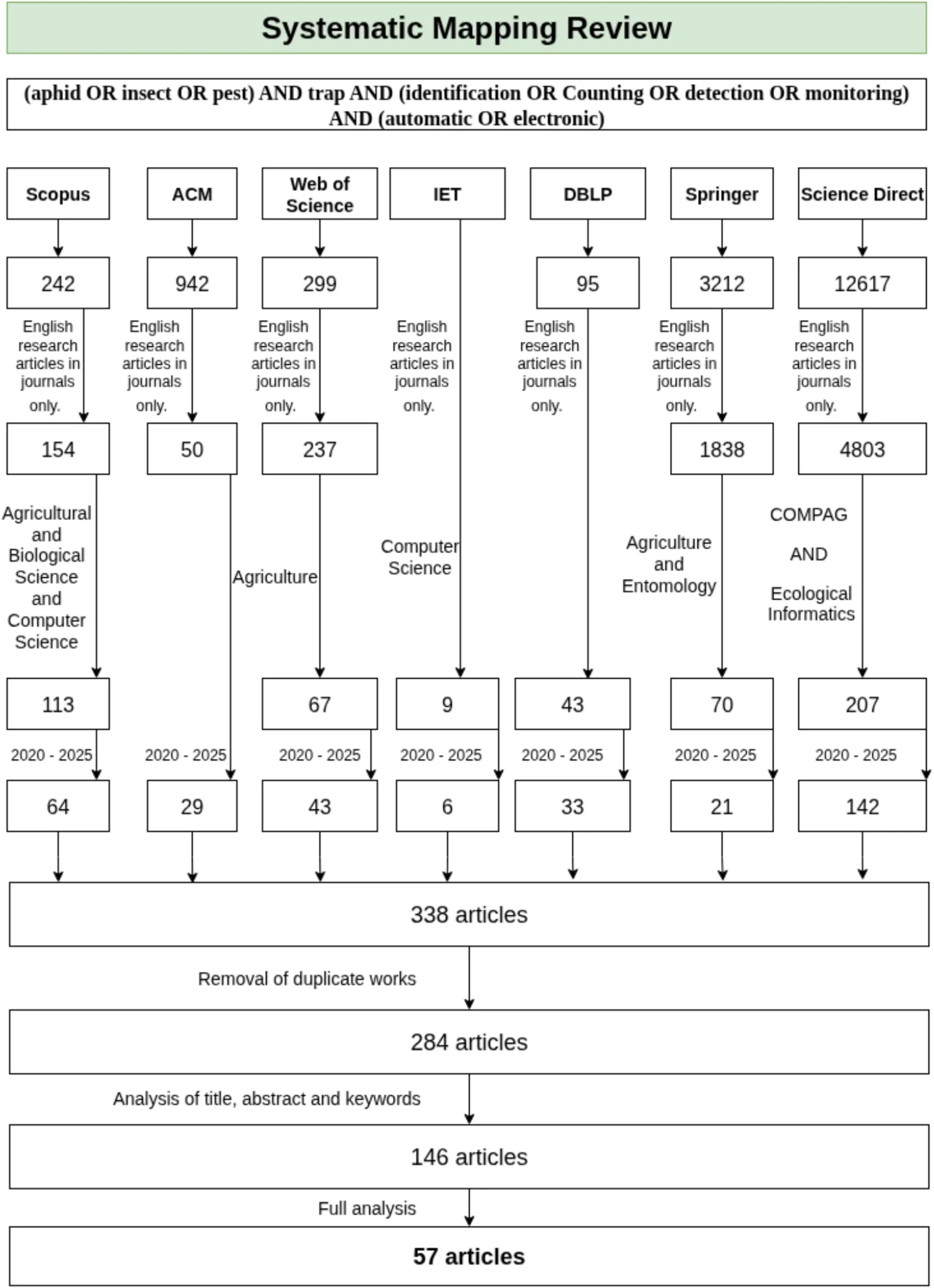
Study selection flowchart for studies in the systematic review

Our review method and eligibility criteria are presented in the “Materials and Methods” section. The “Results” section shows the findings of the analyses in the selected studies. The “Discussion” section discusses and analyzes the approaches found, highlighting the main findings. Finally, the conclusions are presented in the “Conclusions” section.

## Materials and Methods

Our survey covered studies published between 2020 and 2025 and followed the Preferred Reporting Items for Systematic reviews and Meta-Analyses (PRISMA) guidelines (Page et al. 2021). To ensure a comprehensive retrieval of literature at the intersections of agriculture, ecology, and computer science, we consulted seven multidisciplinary and specialized digital databases: Scopus, Association for Computing Machinery (ACM), Web of Science, Institution of Engineering and Technology (IET), Digital Bibliography & Library Project (DBLP), Springer, and Science Direct. Only English-language research articles published in journals were included. The survey and analysis were conducted from May to July 2025. The search strategy was applied to the title, abstract, and keywords metadata fields, ensuring the selection of studies where the core topics were central to the research:

*(aphid OR insect OR pest) AND trap AND (identification OR Counting OR detection OR monitoring) AND (automatic OR electronic)*

Figure 1 illustrates the systematic review process used in this study. In Phase 1, we identified 17,416 records. Phase 2 involved screening for scientific articles written in English and published in journals, resulting in 7082 records. In Phase 3, we applied filters for agriculture, entomology, computer science, and journals focused on these fields, reducing the set to 559 articles. Phase 4 restricted the results to studies published between 2020 and 2025, yielding 338 articles. In Phase 5, we removed duplicates and obtained 284 records. Phase 6 applied the eligibility criteria to the articles retained from Phase 5. This step involved reviewing the title, abstract, and keywords, as well as examining the materials and methods briefly. The criteria were as follows:

- The study must propose an applied computing solution for insect identification and counting.
- Insects must be captured using traps (conventional or electronic) or photographed  in situ directly on plants, under field or laboratory conditions.
- Identification must rely on digital images.

Following the initial screening, 146 studies were selected for full-text review, resulting in 57 articles that met the eligibility criteria for in-depth investigation. These selected studies are detailed in Table S1.

## Results

The 57 studies selected for this research are summarized in Supplementary Material Table S1. This comprehensive list provides the identification and country for each study (first two columns), along with the specific crop and target insect species (third and fourth columns). The fifth column specifies the trap types, ranging from traditional attractants (yellow cards, pheromones, or light traps) to e-traps integrated with attraction methods. The sixth column summarizes the CV and DL methods employed, while the seventh reports on the effective application of IPM, including integration with climate data or prognostic tools.

### Macro-analysis

The 57 selected articles used a wide range of software and hardware technologies to automate pest monitoring in agricultural environments, with an emphasis on digital imaging and DL methods. The studies proposed several image-acquisition alternatives, including digital microscopes, industrial cameras, smartphone cameras, desktop digitizers, and sensors connected to single-board computers. Notably, although the search strategy prominently featured the term “trap,” the eligibility criteria explicitly included studies employing in situ photography directly on plants. This inclusion acknowledges that automated monitoring encompasses both physical trapping systems and direct field observations, provided the objective is the systematic identification and counting of pests.

The studies utilizing physical trapping systems relied on attractants—such as color, pheromones, or light—to draw and retain insects. Within this subset, 20 articles introduced new electronic trap (e-trap) prototypes that integrated imaging sensors and edge computing components, while eight articles used devices developed in earlier research. The remaining studies focused on improving intelligent models for multi-species identification, fine-grained visual classification (FGVC), and generalized counting. These works primarily leveraged public datasets (Xiang et al. 2023; Bollis et al. 2022; Liu et al. 2021) or laboratory-based solutions involving microscopy equipment and industrial cameras (Amarathunga et al. 2022; Ibrahim et al. 2023; Le et al. 2020; De Castro Pereira et al. 2022; Kalfas et al. 2023), often serving as the computational foundation for future in situ or trap-based applications.

In this survey, IPM relevance is categorized based on the application context: most works identify IPM as the motivating framework for automation, while a select subset of studies achieves operational integration by fusing recognition outputs with climatic variables for pest forecasting.

Algorithms such as You Only Look Once (YOLO) variants (YOLOv3, YOLOv4, YOLOv5, YOLOv7, YOLOv8, YOLOv10n), Faster R-CNN, mask R-CNN, EfficientDet, and architectures based on ResNet, MobileNet, and Vision Transformers (ViT, PVTv2, SwinIR) were commonly adapted for recognizing target insects (object detection, classification, and density tasks) (Wang et al. 2025; Hong et al. 2021; De Cesaro Júnior and Rieder 2020; Hechen et al. 2024). These adaptations often included attention mechanisms, such as SENet, CBAM, CA, ECA, SSA, LAHead, and the multi-attention module, to focus on relevant features (Xiang et al. 2023; Bollis et al. 2022; Nanni et al. 2020; Peng and Wang 2022). Feature pyramid networks have also been commonly applied to handle multiple object scales, as well as customizations to loss and anchor functions to optimize the detection of small objects and clusters (Zhang et al. 2024; Liu et al. 2020; Chen et al. 2025a). Additionally, data augmentation and transfer learning techniques were widely employed to improve the robustness and generalizability of the models (Huang et al. 2021; Freitas et al. 2022; Bai et al. 2024).

Regarding solution performance, it is difficult to directly compare results due to differences in datasets, pest classes, imaging conditions, and metrics. However, overall high-performance trends are notable. The most frequently reported metrics were as follows:

- Accuracy: classification task
- Mean average precision (mAP@0.5, mAP@0.5:0.95), precision, recall, and F1-score: object detection task
- Mean absolute error (MAE) and root mean squared error (RMSE): counting tasks
- Coefficient of determination ($R^2$): assessment of regression adjustments

**Fig. 2:**
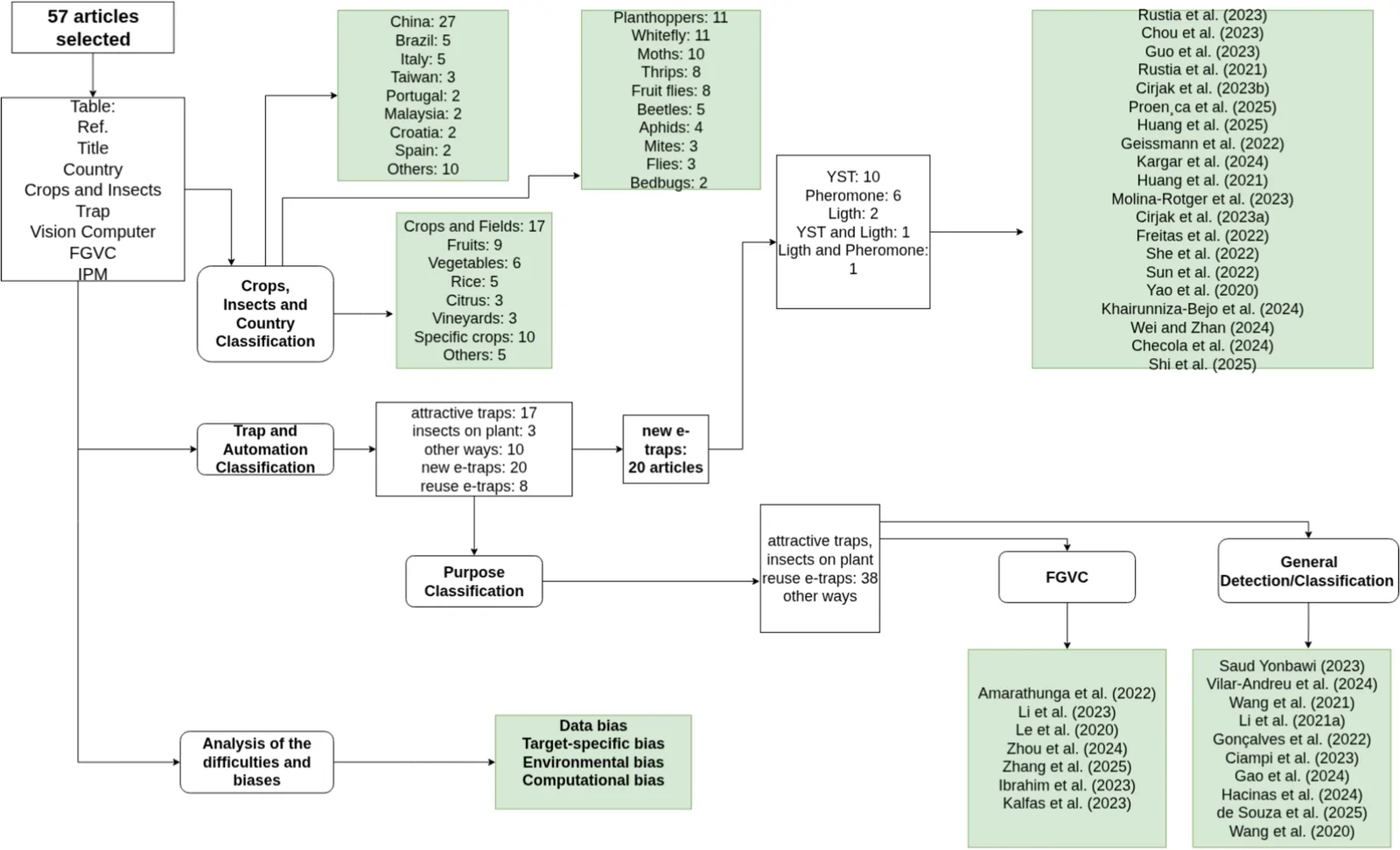
Structured process of systematic literature reviews and respective categorization of results

Figure 2 illustrates a diagram categorized for the analysis of the selected articles, to be explored in the next sections, and evaluated under the following aspects: (i) country, crops, and insect groups; (ii) trap types and automation; (iii) purpose of automatic recognition; and (vi) difficulties and biases reported by the authors.

### Classification by Country, Crops, and Insect Groups

**Fig. 3:**
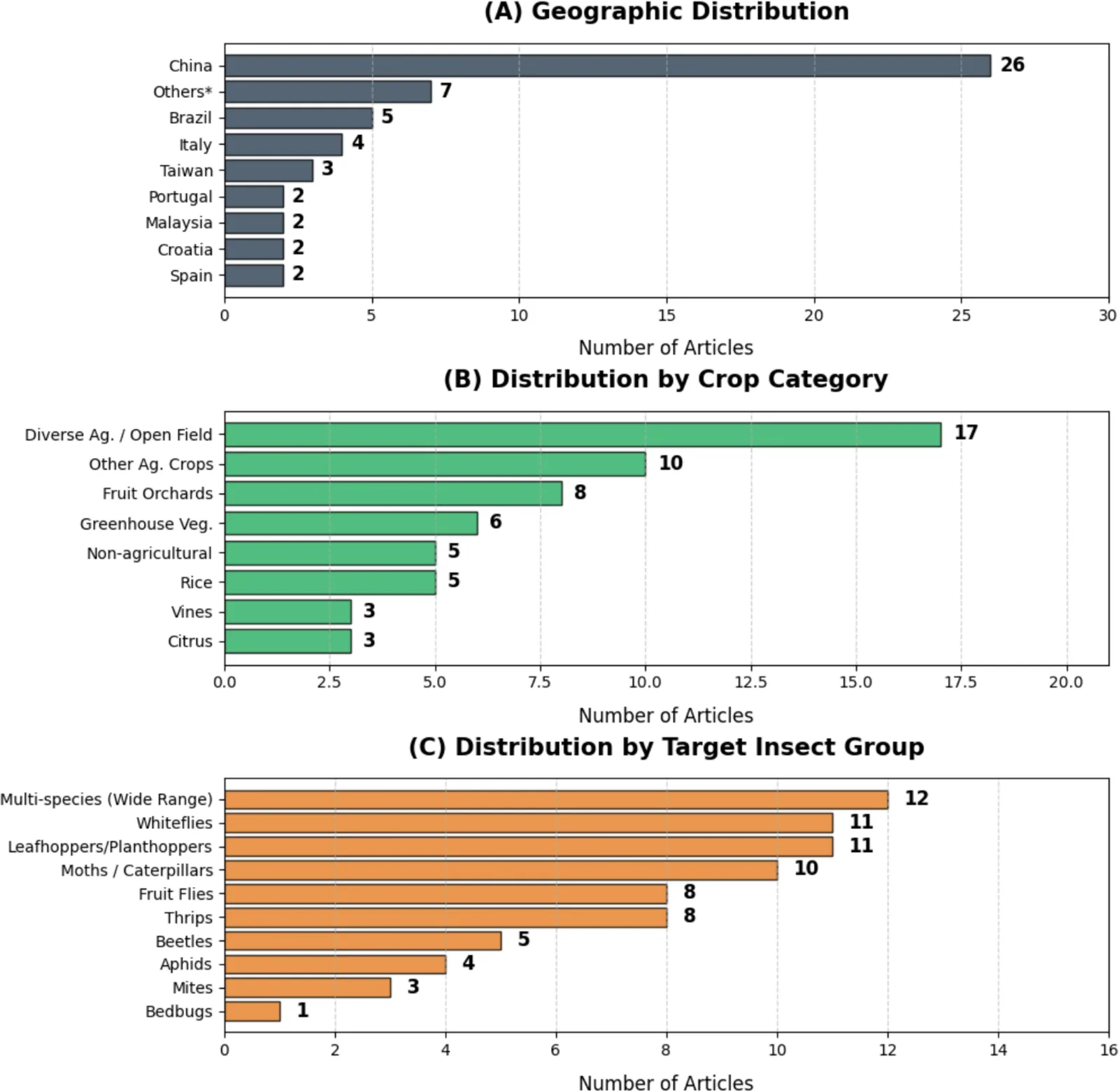
**Quantitative synthesis of the 57 selected studies categorized by geographic, agricultural, and biological dimensions.** **A** Geographic distribution of research articles by country. **B** Distribution of studies by crop category. **C** Frequency of research efforts by the target insect group. Asterisk (*) denotes countries with a single study contribution

The geographic, agricultural, and biological distributions of the selected studies are synthesized in Fig. 3. Panel A reveals a significant geographic concentration of research in China, which accounts for nearly 45% of the total output (*n*=26). To determine the country of origin for this analysis, we considered the primary location of the research group; however, several studies involved international collaboration with researchers from various countries. This dominance is likely driven by China’s massive agricultural production and strategic investments in Agriculture 4.0 and early warning technologies (Wu et al. 2022), resulting in leading research centers and the creation of pivotal public datasets, exemplified by Pest24 and IP102. Other significant contributions come from Brazil (*n*=5), Italy (*n*=4), and Taiwan (*n*=3), where research is often tailored to locally relevant economic crops such as fruits and vegetables.

Regarding the agricultural context (Fig. 3B), research is primarily divided between diverse field crops (*n*=17) and fruit orchards (*n*=8), with a notable presence in greenhouse-grown vegetables (*n*=6) and rice (*n*=5).

The target insect groups (Fig. 3C) further illustrate the field’s economic priorities. While many studies (*n*=12) focus on broad-spectrum pest detection using generalist datasets, specific research efforts are concentrated on leafhoppers and planthoppers (*n*=11), whiteflies (*n*=11), and moths or caterpillars (*n*=10).

### Classification by Trap Types and Automation

All studies that used traps to capture insects employed attractants, including yellow color, light, and sex pheromones. No active mechanisms, such as suction, were applied. Ten studies used yellow sticky traps (YSTs) or yellow trays with an aqueous solution (Moericke type) (Wang et al. 2024; Ciampi et al. 2023; Zhang et al. 2024; Bai et al. 2024; Gao et al. 2024; Gonçalves et al. 2022; Amarathunga et al. 2022; De Cesaro et al. 2022; Sun et al. 2024; Kalfas et al. 2023). They have been conventionally exposed, with the imaging or monitoring process still involving a manual intervention for card collection and laboratory digitization. Two studies used pheromones (De Souza et al. 2025; Hong et al. 2021), and four others applied combined attractants (Zhang et al. 2022; Ibrahim et al. 2023; Li et al. 2023; Hacinas et al. 2024).

Additionally, 13 studies (22.8%) were also captured by the search criteria despite not using physical traps. Among these, three studies focused on capturing images of insects directly on the host plant (Zhou et al. 2024; De Castro Pereira et al. 2022; Zhang et al. 2025). In these cases, the plant functions as the monitoring substrate, presenting a highly challenging scenario for CV: high variability in natural lighting, unconstrained and varied backgrounds, and the need for fine focus adjustment on live specimens. The other ten studies used specialized datasets or laboratory images to extract subtle morphological features (Le et al. 2020; Peng and Wang 2022; Liu et al. 2021, 2020; Hechen et al. 2024; Vilar-Andreu et al. 2024; Nanni et al. 2020; Yonbawi et al. 2023; Bollis et al. 2022; Xiang et al. 2023). The inclusion of these studies is essential to enrich the systematic review, as they address the core requirements of FGVC under realistic conditions, providing the high-fidelity data necessary to train robust models for next-generation smart traps.

Twenty studies presented electronic trap (e-traps) prototypes to gather real-time data. They integrated electronic components, such as sensors, wireless communication boards, single-board computers, microcontrollers, and photovoltaic panels, among other resources, to automate image acquisition in the field, as well as data processing and transmission. In these devices, attractants were used to attract insects. These studies may be categorized according to the attractant used, as follows:

- YSTs: ten articles (Huang et al. 2025; Geissmann et al. 2022; Proença et al. 2025; Rustia et al. 2023; Chou et al. 2023; Kargar et al. 2024; Guo et al. 2023; Rustia et al. 2021; Checola et al. 2024; Huang et al. 2021)
- Sex pheromones: six articles (Molina-Rotger et al. 2023; Čirjak et al. 2023a, b; Freitas et al. 2022; She et al. 2022; Wei and Zhan 2024);
- Light: two articles (Sun et al. 2022; Yao et al. 2020)
- YST and light: one article (Khairunniza-Bejo et al. 2024)
- Light and pheromone: one article (Shi et al. 2025)

This survey demonstrates that YSTs were the most employed. The availability of pheromones for certain species and the field power generation for light emission render these options more challenging to implement, making them less widespread. Regarding automation, the study by Huang et al. (2021) stands out for presenting an e-trap prototype with a motorized system, which automatically rotates YSTs based on the saturation level of the adhesive surface.

Regarding the recognition of target insects, the best performances were observed in prototypes that transmit the digital images to remote/cloud servers (Wei and Zhan 2024; Khairunniza-Bejo et al. 2024; She et al. 2022). These locations have an increased availability of computational resources, such as graphics processing units (GPUs) that enable image prediction on more complex and larger DL models. However, this alternative requires implementing an appropriate communication network that takes into account image size and sending frequency.

Another strategy explored by some authors is image prediction on the device (edge computing), commonly implemented in remote locations with limited or intermittent connectivity or to minimize energy consumption and operational costs. Considering these objectives, edge device models are often lighter and smaller, with fewer parameters and floating-point operations (FLOPs). In some cases, this may imply a slight compensation for maximum accuracy compared to conventional one- or two-stage models running on servers with high graphics processing power via GPUs. Models such as YOLOv8-Nano/Small, TinyYOLOv3, and SCA-YOLOv5s, together with study-specific optimizations (e.g., a 2.4 MB model size and 5.6 ms processing time reported in Wei and Zhan (2024)), demonstrate this perception.

It is worth noting that “best performance” may be subjective depending on whether the priority is maximum accuracy, speed, edge device efficiency, or the ability to handle complex conditions in the field. In summary, although higher performance values in some raw metrics still appear in systems with remote processing, advances in local processing of edge devices are providing very high accuracies. These values are more than sufficient for practical pest monitoring applications, especially when speed, autonomy, and resource efficiency are critical factors.

Regarding edge computing applications, Raspberry Pi (Zero W and 4B series) single-board computers stand out for running optimized models. This context highlights (Čirjak et al. 2023a), who performed tests that achieved accuracy and F1-scores close to 100% for detecting apple moth using a Raspberry Pi 4B. Rustia et al. (2023) achieved an F1-score of 0.96 with local processing on a Raspberry Pi Zero W. Other studies, such as Freitas et al. (2022) and Kargar et al. (2024), demonstrate accuracy values above 90% with local processing, prioritizing efficiency.

The electronic trap prototypes proposed in these studies are predominantly in the early validation phase, which reflects the limited large-scale deployment and integration of electronic trap networks into current agricultural monitoring. This assessment is supported by the scale of implementation and duration of experiments detailed in the Supplementary Material Table S4. The prevalence of projects utilizing only one or two units confirms that the primary focus remains on validating CV technology and low-cost edge computing hardware, rather than systemic network deployment or volume production. This “Evolving” status is further evidenced by the duration of the studies, which suggests an incipient or short-term validation nature in most cases. The majority of validations span from seven days to six months, a timeframe insufficient to cover multiple seasonal pest cycles or demonstrate long-term robustness under varying climatic conditions (a critical step for progression toward effective IPM decision support). Notably, only Rustia et al. (2023) report a 2-year validation, indicating a more mature system in terms of field application longevity.

### Classification by Automatic Recognition Purpose

The other 38 articles explored different techniques for automatic pest recognition in contexts that may be later integrated into monitoring systems or laboratory strategies. These studies were categorized into two research fronts based on their recognition depth and hardware requirements. Technical specifications, architectures, and metrics for both groups are detailed in the Supplementary Material Tables S2 and S3. The research fronts are as follows:

- **Group A (FGVC):** comprises studies extracting subtle morphological characteristics of minute species with high inter-class similarity or different developmental stages
- **Group B (generic detection and classification):** focuses on identifying broad classes for operational monitoring without delving into subtle taxonomic distinctions

Group A reflects a significant effort to achieve species-level identification through architectural modifications designed to capture high-order correlations. Part-based and attention-driven strategies are the primary solutions identified. For instance, explicit physical segmentation of the insect body (head, thorax, and abdomen) is combined with stacked ViT to analyze discriminative regions (Amarathunga et al. 2022). Complementarily, unsupervised spatial and channel attention mechanisms, such as Coordination and Local Attention (CLA) in AgriPest-YOLO (Zhang et al. 2022), Pairwise Self-Attention (PSA) in ASP-Det (Wang et al. 2022), and the Convolutional Block Attention Module (CBAM) in Insect-YOLO (Wang et al. 2025), act as implicit part detectors. These modules refine the location of characteristics (e.g., wing venation or tarsal structures), as seen in the Coordinate Attention (CA) of SCA-YOLOv5s (Sun et al. 2024), effectively reducing visual interference from background noise (Liu et al. 2021; Bai et al. 2024; Xiang et al. 2023; Chen et al. 2025b).

The adoption of transformers and hybrid architectures (ViT + CNN) further enhances this front by balancing global contextual information with the efficiency of convolutional layers. This is evidenced by backbones like the Pyramid Vision Transformer (PVTv2) in Pest-PVT (Chen et al. 2025a), custom mechanisms in DWViT-ES (Hechen et al. 2024), and the integration of ConvNeXt or CIE blocks into YOLO frameworks to enhance feature extraction at multiple scales (Xiang et al. 2023; Wang et al. 2024; Peng and Wang 2022). Additionally, multistage and cascade classifiers are utilized to progressively refine detection, moving from high-resolution feature extraction with Mask R-CNN (De Cesaro et al. 2022) to tree-based classifiers for fine configuration (Rustia et al. 2021, 2023; De Castro Pereira et al. 2022).

Beyond standard algorithms, specialized hardware and complex databases are driving FGVC. High-fidelity acquisition through microscopy, industrial cameras, or scanners (Amarathunga et al. 2022; Li et al. 2023) is essential for distinguishing species like *Liriomyza* spp. or microscopic mites (Supplementary Material Table S5). Furthermore, the use of challenging datasets such as IP102 and Pest24 (characterized by class imbalance and visual overlap) drives the development of salience-based preprocessing (Nanni et al. 2020) and detailed manual or point annotations to handle densely distributed objects (Zhang et al. 2025; Ibrahim et al. 2023; Ciampi et al. 2023).

Group B prioritizes operational efficiency for large-scale counting. The YOLO family is the dominant choice (v3, v4, and v8) due to its trade-off between speed and precision on edge devices (De Souza et al. 2025; Hacinas et al. 2024; Wang et al. 2021). While Group A seeks taxonomic depth, Group B focuses on density robustness. Studies in this group consistently achieve high correlation with manual counting, with $R^2$ values above 0.97 even in high-density scenarios (Liu et al. 2021; Hacinas et al. 2024; De Souza et al. 2025). Notably, generalist approaches have successfully achieved mAP values up to 96.7% by grouping diverse species into single operational categories (Vilar-Andreu et al. 2024) or employing hybrid MMTL-IPCAC approaches for high-precision classification (Yonbawi et al. 2023).

## Discussion

Our findings confirm that the 2020–2025 period was marked by the consolidation of the YOLO series as the standard for real-time detection and the emergence of architectures based entirely on transformers, such as the ViT. Unlike the incipient attention mechanisms integrated into CNNs seen in the previous quinquennium, these new architectures are based purely on self-attention mechanisms, allowing for more effective capture of global dependencies in pest images. In parallel, the availability of high-performance edge computing devices has effectively enabled the transition from theoretical models to the autonomous embedded solutions and smartphone-based digital scouting applications discussed in this review.

With the increasing availability and processing power of smartphones, these devices have emerged as accessible and versatile tools for pest monitoring in agricultural systems. Recent studies have investigated their potential through both hardware adaptations and advanced on-device data processing techniques. For instance, Li et al. (2023) developed an intelligent field investigation system that employs a macro lens (200× magnification) and an adjustable support attached to the smartphone camera, enabling the acquisition of high-quality images of microscopic insects, such as species of the genus Liriomyza, in their natural habitats. In a different approach, Zhou et al. (2024) presented a mobile application capable of performing autonomous pest detection directly on the device. The application integrates the YOLOv7-tiny deep learning model to identify and count pests in real time without requiring internet connectivity, thereby overcoming common connectivity constraints in rural areas. Additionally, it generates spatial distribution maps that support site-specific pesticide applications.

**Fig. 4:**
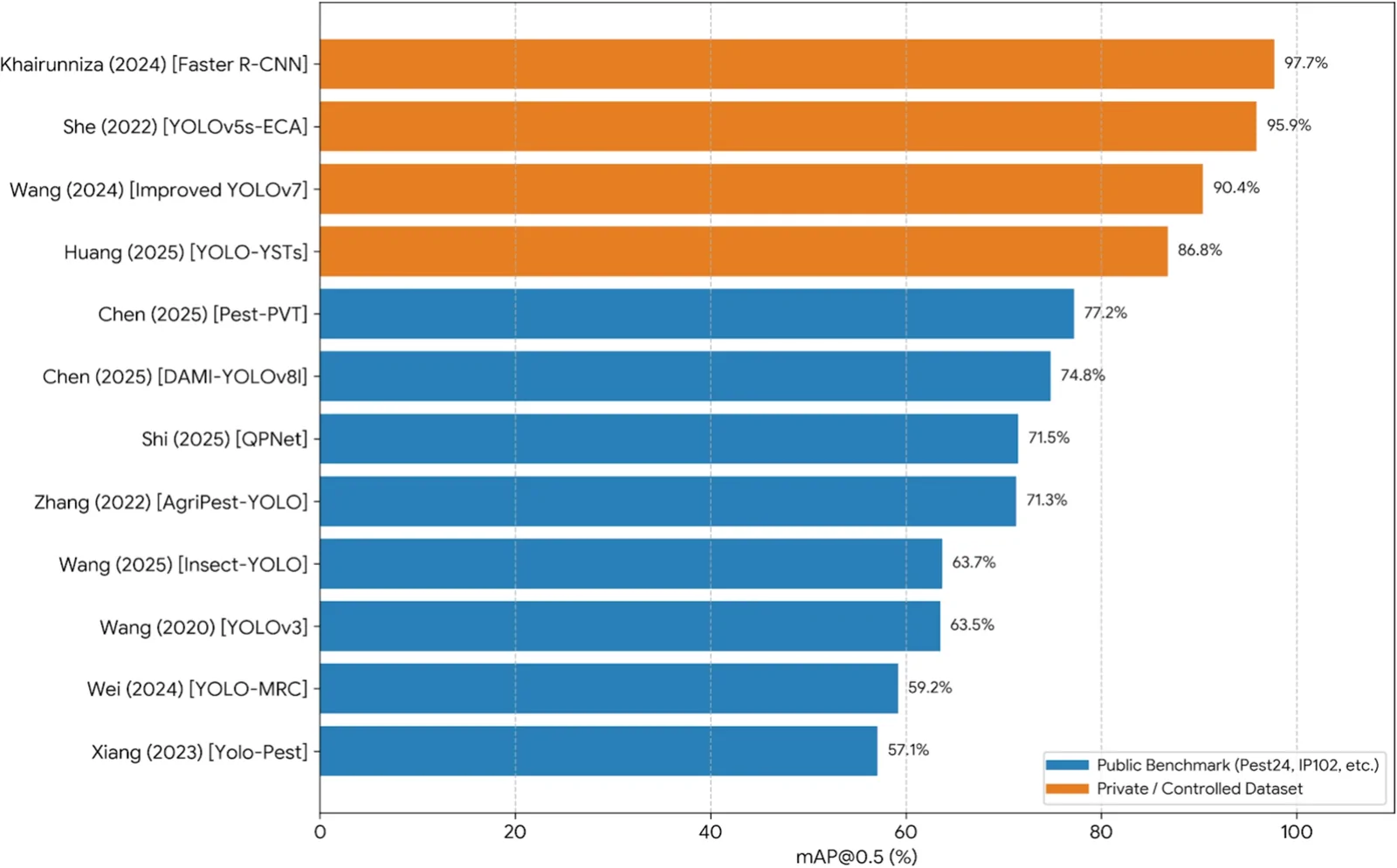
Comparative performance (mAP@0.5) of DL models across different dataset types. Studies utilizing complex public benchmarks (e.g., Pest24, IP102) show significantly lower precision due to environmental noise and multi-class overlap, while those based on controlled private datasets consistently achieve results above 85%, highlighting the gap between experimental validation and real-world deployment challenges

Another important point concerns the complexity associated with monitoring processes. In this context, uncertainty in automated monitoring can be categorized into three dimensions: target-specific, environmental, and computational. Target-specific bias arises from the minute size and high morphological similarity of species, necessitating FGVC and high-magnification hardware (Amarathunga et al. 2022; Wang et al. 2020; Chen et al. 2025a). Environmental bias, also referred to as contextual noise, arises from variable lighting conditions, debris, and insect overlap in field traps, degrading image quality and increasing the incidence of false positives (De Cesaro et al. 2022; Checola et al. 2024; Rustia et al. 2021; Wang et al. 2020). Finally, computational bias relates to the scarcity of balanced, large-scale public datasets and the inherent trade-offs between model complexity and the limited processing power of edge devices (Hechen et al. 2024; Liu et al. 2021).

Regarding the distribution of studies across crops, the versatility of CV systems becomes evident, as they are being validated across a wide range of environments, from intensive greenhouse production to extensive open-field farming. The use of multi-species datasets in these contexts highlights a trend toward models designed to address the high biodiversity found in real-world agricultural ecosystems. This aspect is particularly relevant for primary crop pests and pathogen vectors affecting high-value crops, which require high-frequency monitoring to support effective IPM. Detailed references for each country, crop, and insect species mentioned are provided in Supplementary Material Table S1.

The availability of complex public datasets, such as IP102, Pest24, and SA-1B, complemented by extensive proprietary data, has been pivotal for model training. However, a significant performance gap is observed depending on the dataset complexity. While studies utilizing the Pest24 public dataset achieve a maximum mAP@0.5 of approximately 77.2% (Chen et al. 2025a), due to high occlusion and multi-class complexity, models trained on private datasets often report superior results, exceeding 90% mAP@0.5 (Khairunniza-Bejo et al. 2024; She et al. 2022). This discrepancy, detailed in the Supplementary Material Table S6, and summarized in Fig. 4, highlights the challenge of maintaining high precision in unconstrained public benchmarks compared to more controlled private environments. The lower performance in public sets underscores the ongoing need for more robust architectures to handle field-level complexity, as emphasized in the “Classification by Automatic Recognition Purpose” section.

Furthermore, the construction of high-quality datasets is hindered by the “taxonomic bottleneck,” as precise annotation of fine-grained features requires high-level expertise, making the scaling of automated systems a labor-intensive process (Checola et al. 2024). Addressing these multi-layered uncertainties is critical for ensuring the transferability of models from controlled datasets to real-world agricultural ecosystems.

To mitigate data scarcity and class imbalance, recent literature has begun to explore the use of Generative AI (GANs and diffusion models) as a complementary strategy to traditional augmentation, enabling the generation of high-fidelity synthetic samples for rare or underrepresented pest classes (Karam et al. 2022; Du et al. 2025). This approach offers an effective means of addressing the “data bias” identified in earlier studies, enabling more robust model training without the logistical costs associated with extensive field data collection.

Automatic identification, in most cases, requires CNNs for training the smart models. The primary task of these models is object detection, followed by image classification and the density maps that enable insect counting. Among CNN-based applications, the YOLO family and its variants (YOLOv3, YOLOv4, YOLOv5, YOLOv7, YOLOv8, YOLOv10) are the most widely employed. According to Hakim et al. (2025), these models are preferred due to their favorable balance between inference speed and detection accuracy.

The growing emphasis on FGVC addresses the challenge of distinguishing species or developmental stages with minimal morphological differences (such as whitefly instars, thrips, or visually similar planthoppers) (Hanselmann and Ney 2020). Technically, the distinction between standard object detection and true FGVC is rooted in architectural modifications designed to capture these subtle inter-class differences. Beyond standard attention modules, reviewed SOTA models utilize strategies such as high-order feature correlations (e.g., bilinear pooling) and part-based localization to focus on discriminative traits like wing venation or tarsal structures (Chen et al. 2025a; Amarathunga et al. 2022). These approaches allow the network to suppress unconstrained background noise while amplifying fine-scale pixel dependencies, a requirement that standard YOLO backbones often struggle to meet without specialized refinement layers. Complementarily, studies leverage microscopy or high-resolution cameras with macro lenses to capture the high-fidelity morphological characters essential for these precise taxonomic distinctions (Amarathunga et al. 2022; Zhou et al. 2024; Le et al. 2020; Li et al. 2023).

Regarding hardware and microscopy resources for digital image acquisition, the 28 studies addressing FGVC predominantly employed enhanced imaging setups, including high-resolution industrial cameras (e.g., 24-megapixel Fujifilm X-T1 and 6-megapixel HIK Vision MV-CA060-10GC). Microscopy resources, including magnifying lenses, portable digital microscopes (Dino-Lite) used by Amarathunga et al. (2022), and trinocular microscopes, enabled the visualization of subtle morphological characteristics in extremely small specimens. Microscopy may be an alternative to capturing fine details of tiny insects, allowing DL architectures to extract and focus on local and discriminating characteristics, generating models capable of detecting and classifying tiny pests.

The integration of attention mechanisms, the emergence of transformer-based and hybrid CNN–ViT architectures, and the adoption of multistage pipelines, including cascade classifiers, constitute dominant trends. While CNNs are the backbone for extracting local characteristics, transformers capture long-range dependencies and the global context (Shah and Tembhurne 2023; Maurício et al. 2023). These innovations, combined with fuller datasets and, when necessary, high-magnification imaging resources (microscopy/ industrial cameras), enable models to overcome the FGVC challenges of detecting and classifying pests with high accuracy, even when considering subtle visual distinctions.

Concerning automatic pest monitoring via e-traps, 20 studies (35%) proposed DL-based solutions based on edge computing to enable practical field deployment. While these studies demonstrate critical optimizations for on-device inference (such as model quantization, depth-separable convolutions, and lightweight backbones including ShuffleNetv2 Sun et al. 2024; Xiang et al. 2023), large-scale operational deployment and integration of these networks into IPM systems remain a significant bottleneck. Current research is primarily characterized by isolated prototypes focusing on individual device performance rather than the systemic reliability required for long-term field monitoring. This reflects a persistent computational bias where the trade-off between high taxonomic precision and the hardware constraints of single-board computers or microcontrollers must be carefully balanced to ensure real-time analysis (Hechen et al. 2024; Liu et al. 2021).

Furthermore, the transition from automated counting to decision support for pest management systems is, in many cases, still in its early stages. The practical use of this information requires a multidisciplinary approach. Count data should be transmitted to a database and used as input in population growth simulation models. This model needs to be integrated with a climate forecasting model, host development models, and be able to establish the relationship between the predicted pest population and the damage caused, in order to assess whether economic damage thresholds will be reached. Although most articles acknowledge the significance of IPM, explicit climate data cross-referencing—essential for outbreak prediction—is less common, appearing in only four studies (Rustia et al. 2023; Shi et al. 2025; Huang et al. 2021; Chou et al. 2023). This integration is not merely a software challenge but a complex interdisciplinary requirement involving the acquisition, processing, and analysis of large volumes of biological and environmental data.

Beyond computational performance, the large-scale adoption of smart traps by growers is hindered by critical socio-economic and infrastructural barriers that move the challenge from the laboratory to the field. First, economic viability remains a decisive factor; while traditional sticky traps are low-cost, the initial investment in electronic stations is significantly higher. As noted by Chou et al. (2023), the high cost is driven not only by sensor installation and wireless infrastructure but also by the professional knowledge required for long-term maintenance of AIoT systems. Literature suggests that system viability depends on minimizing per-unit costs to enable the high-density deployment required in orchards and large-scale cropping systems (Kargar et al. 2024). Furthermore, the field still lacks robust quantification of the return on investment (ROI) derived from pesticide savings, which is essential to convince risk-averse producers.

**Fig. 5:**
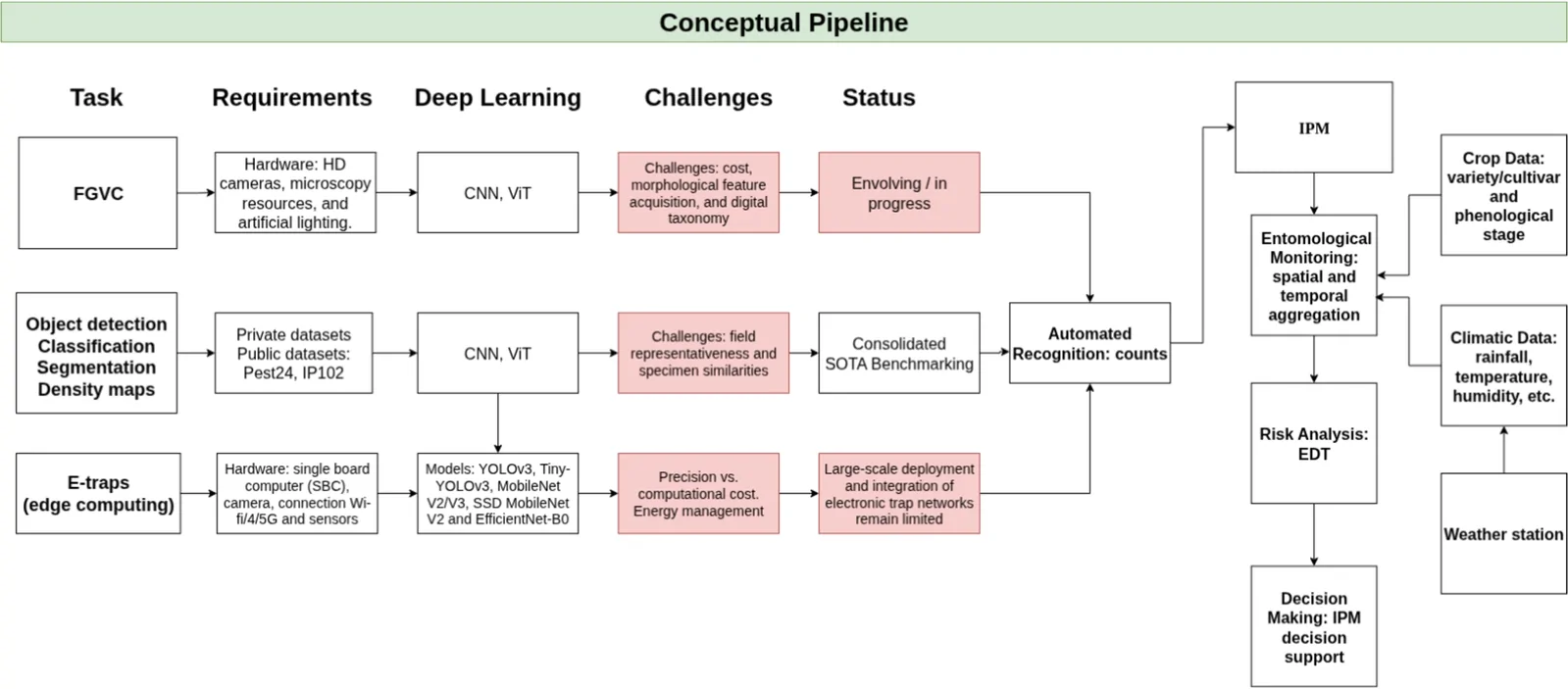
Conceptual pipeline from automated recognition to IPM decision support. The pipeline integrates FGVC, detection models, and e-traps with entomological monitoring and EDT-based risk analysis. The red-shaded components in the “Challenges” and “Status” columns indicate processes that are not yet consolidated in current research and operational practice

Second, connectivity gaps in rural areas represent a systemic bottleneck, as many agricultural zones are located far from urban centers with reliable internet infrastructure (Kargar et al. 2024). To overcome this, recent studies emphasize the necessity of full edge computing autonomy. Developing algorithms specifically optimized for edge devices allows for accurate detection and recognition even without a stable internet connection (Rustia et al. 2023). This shift toward on-site processing also enables the transmission of results using minimal data bytes, ensuring that the system remains functional in areas with limited bandwidth (Čirjak et al. 2023b).

Third, the transition toward effective IPM depends on multimodal data integration, specifically the inclusion of local meteorological sensors. Relying on remote weather data is often insufficient for predicting local outbreaks, as microclimatic variables directly influence pest life cycles and economic damage thresholds (EDT). Integrating real-time parameters such as air temperature and humidity in the specific spraying area is critical for determining the optimal moment and duration of chemical interventions (Molina-Rotger et al. 2023). Furthermore, as highlighted by Chou et al. (2023), combining daily pest counts with soil conditions and ambient metrics enables more accurate forecasting. However, most current systems do not capture hyper-local environmental variables, including canopy-level rainfall and wind speed, which are essential for identifying pest hotspots and predicting within-field dispersal patterns (Rustia et al. 2023).

Finally, these limitations indicate that autonomous monitoring stations should be designed not as isolated camera modules, but as robust, self-sufficient units. To be operationally viable, they must integrate energy harvesting with localized intelligence and multimodal sensing, bridging the gap between computational complexity and the practicalities of a sustainable, field-ready IPM strategy.

To synthesize these findings and address the identified gaps, we propose a conceptual pipeline (Fig. 5) that delineates the necessary progression from automated recognition to actionable IPM. This pipeline illustrates how the technical outputs from the three primary research fronts—FGVC, SOTA benchmarking, and e-traps—serve as the technical foundation for automated recognition. However, as shown in the framework, true maturation occurs only when these counts are integrated into an entomological monitoring phase. In this stage, counts are contextualized by monitoring metadata, including georeferencing and historical series, which are essential for calculating spatial and temporal aggregations. This monitoring is further enriched by multimodal integration with crop data (e.g., variety/cultivar and phenological stage) and climatic variables retrieved from local weather stations. This structured workflow culminates in a risk analysis grounded in simulation and forecasting models (EDT), providing the definitive intelligence required for effective decision support in field interventions.

## Conclusions

This survey analyzed the evolution of DL applied to pest recognition between 2020 and 2025, revealing a significant shift from generic classification toward FGVC and hybrid CNN-Transformer architectures. While technical accuracy has reached impressive benchmarks on controlled datasets, its operational deployment in real-world Agriculture 4.0 scenarios still faces critical hurdles.

Several substantive gaps in the literature remain to be addressed: (i) the performance gap between controlled datasets and unconstrained field conditions, where environmental noise and high specimen density significantly degrade model reliability; (ii) the limited large-scale deployment of electronic trap networks, which remain mostly as localized prototypes; and (iii) the insufficient integration of automated counts with climatic variables and EDT, limiting the transition from simple recognition to actionable decision support in IPM.

To overcome these barriers, future research should prioritize the following:

- **Data enrichment:** leveraging generative AI, such as GANs and diffusion models, to mitigate the “taxonomic bottleneck” and address data scarcity for rare or minute species
- **Edge computing optimization:** developing lightweight and energy-efficient models (e.g., through quantization and pruning) that maintain high taxonomic precision on resource-constrained devices
- **Multimodal integration:** incorporating real-time environmental data from local meteorological sensors, aligning CV outputs with entomological theory for outbreak prediction
- **Standardization and economic validation:** establishing rigorous field-testing protocols and quantifying the return on investment (ROI) to facilitate broader adoption by the agricultural sector

Furthermore, while our search window captures the rise of hybrid architectures, recent developments point to the next methodological steps in the field. In particular, the emergence of models that eliminate the traditional non-maximum suppression (NMS) step, most notably in YOLOv10 and YOLO26 (Chakrabarty 2026), alongside architectural refinements in YOLOv9 and YOLOv11, illustrates this ongoing evolution. By natively bypassing the NMS bottleneck, these architectures offer promising improvements in parameter efficiency and in the detection of heavily occluded or clustered specimens.

Ultimately, the path toward fully automated and sustainable pest monitoring hinges on bridging the gap between computational complexity and field robustness. The proposed conceptual pipeline provides a roadmap for this evolution, ensuring that the next generation of smart traps delivers not merely counts, but the essential intelligence required for effective IPM.

## Supplementary Information

Below is the link to the electronic supplementary material.

Supplementary file 1 (pdf 212 KB)

## Data Availability

All data generated or analysed during this study are included in this published article and its supplementary information files.

## References

[CR1] Amarathunga DC, Ratnayake MN, Grundy J et al (2022) Fine-grained image classification of microscopic insect pest species: Western flower thrips and plague thrips. Comput Electron Agric 203:107462. 10.1016/j.compag.2022.107462

[CR2] Bai M, Chen T, Yuan J et al (2024) A point-based method for identification and counting of tiny object insects in cotton fields. Comput Electron Agric 227:109648. 10.1016/j.compag.2024.109648

[CR3] Bollis E, Maia H, Pedrini H et al (2022) Weakly supervised attention-based models using activation maps for citrus mite and insect pest classification. Comput Electron Agric 195:106839. 10.1016/j.compag.2022.106839

[CR4] Chakrabarty S (2026) Yolo26: An analysis of NMS-free end to end framework for real-time object detection. arXiv preprint arXiv:2601.12882.

[CR5] Checola G, Sonego P, Zorer R, Mazzoni V, Ghidoni F, Gelmetti A, Franceschi P (2024) A novel dataset and deep learning object detection benchmark for grapevine pest surveillance. Front Plant Sci 15:1485216. 10.3389/fpls.2024.1485216 PMC11669504 39726421

[CR6] Chen H, Wen C, Zhang L et al (2025) Pest-PVT: a model for multi-class and dense pest detection and counting in field-scale environments. Comput Electron Agric 230. 10.1016/j.compag.2024.109864

[CR7] Chen X, Yang X, Hu H et al (2025) DAMI-YOLOV8l: a multi-scale detection framework for light-trapping insect pest monitoring. Eco Inform 86. 10.1016/j.ecoinf.2025.103067

[CR8] Chou CY, Chang SC, Zhong ZP et al (2023) Development of AIoT system for facility asparagus cultivation. Comput Electron Agric 206. 10.1016/j.compag.2023.107665

[CR9] Ciampi L, Zeni V, Incrocci L et al (2023) A deep learning-based pipeline for whitefly pest abundance estimation on chromotropic sticky traps. Eco Inform 78:102384. 10.1016/j.ecoinf.2023.102384

[CR10] De Castro Pereira R, Hirose E, Ferreira de Carvalho OL et al (2022) Detection and classification of whiteflies and development stages on soybean leaves images using an improved deep learning strategy. Comput Electron Agric 199:107132. 10.1016/j.compag.2022.107132

[CR11] De Cesaro Júnior T, Rieder R (2020) Automatic identification of insects from digital images: a survey. Comput Electron Agric 178. 10.1016/j.compag.2020.105784

[CR12] De Cesaro JT, Rieder R, Di Domênico JR et al (2022) InsectCV: a system for insect detection in the lab from trap images. Eco Inform 67. 10.1016/j.ecoinf.2021.101516

[CR13] Du M, Wang F, Wang Y et al (2025) Improving long-tailed pest classification using diffusion model-based data augmentation. Comput Electron Agric 234:110244. 10.1016/j.compag.2025.110244

[CR14] Freitas L, Martins V, de Aguiar M, et al (2022) Deep learning embedded into smart traps for fruit insect pests detection. ACM Trans Intell Syst Technol 14(1). 10.1145/3552435

[CR15] Gao X, Xue W, Lennox C et al (2024) Developing a hybrid convolutional neural network for automatic aphid counting in sugar beet fields. Comput Electron Agric 220:108910. 10.1016/j.compag.2024.108910

[CR16] Geissmann Q, Abram PK, Wu D et al (2022) Sticky pi is a high-frequency smart trap that enables the study of insect circadian activity under natural conditions. PLoS Biol 20(7):1–26. 10.1371/journal.pbio.3001689 PMC9262196 35797311

[CR17] Gonçalves J, Silva E, Faria P, et al (2022) Edge-compatible deep learning models for detection of pest outbreaks in viticulture. Agronomy 12(12). 10.3390/agronomy12123052

[CR18] Guo Q, Wang C, Xiao D et al (2023) Automatic monitoring of flying vegetable insect pests using an rgb camera and yolo-sip detector. Precision Agric 24(2):436–457. 10.1007/s11119-022-09952-w

[CR19] Hacinas EAS, Querol LS, Santos KLT, et al (2024) Rapid automatic cacao pod borer detection using edge computing on low-end mobile devices. Agronomy 14(3). 10.3390/agronomy14030502

[CR20] Hakim A, Srivastava AK, Hamza A et al (2025) Yolo-pest: an optimized yolov8x for detection of small insect pests using smart traps. Sci Rep 15(1):14029. 10.1038/s41598-025-97825-3 PMC12019348 40269001

[CR21] Hanselmann H, Ney H (2020) Fine-grained visual classification with efficient end-to-end localization. arXiv preprint arXiv:2005.05123.

[CR22] Hechen Z, Huang W, Yin L et al (2024) Dilated-windows-based vision transformer with efficient-suppressive-self-attention for insect pests classification. Eng Appl Artif Intell 127:107228. 10.1016/j.engappai.2023.107228

[CR23] Hong SJ, Nam I, Kim SY, et al (2021) Automatic pest counting from pheromone trap images using deep learning object detectors for matsucoccus thunbergianae monitoring. Insects 12(4). 10.3390/insects12040342 PMC8068825 33921492

[CR24] Huang R, Yao T, Zhan C, et al (2021) A motor-driven and computer vision-based intelligent e-trap for monitoring citrus flies. Agriculture 11(5). 10.3390/agriculture11050460

[CR25] Huang Y, Liu Z, Zhao H, et al (2025) Yolo-ysts: An improved yolov10n-based method for real-time field pest detection. Agronomy 15(3). 10.3390/agronomy15030575

[CR26] Ibrahim MF, Khairunniza-Bejo S, Hanafi M, et al (2023) Deep cnn-based planthopper classification using a high-density image dataset. Agriculture 13(6). 10.3390/agriculture13061155

[CR27] Čirjak D, Aleksi I, Lemic D, et al (2023a) Efficientdet-4 deep neural network-based remote monitoring of codling moth population for early damage detection in apple orchard. Agriculture 13(5). 10.3390/agriculture13050961

[CR28] Čirjak D, Aleksi I, Miklečić I, et al (2023b) Monitoring system for leucoptera malifoliella (o. costa, 1836) and its damage based on artificial neural networks. Agriculture 13(1). 10.3390/agriculture13010067

[CR29] Kalfas I, De Ketelaere B, Bunkens K et al (2023) Towards automatic insect monitoring on witloof chicory fields using sticky plate image analysis. Eco Inform 75:102037. 10.1016/j.ecoinf.2023.102037 PMC10295114 37397435

[CR30] Karam C, Awad M, Abou Jawdah Y et al (2022) Gan-based semi-automated augmentation online tool for agricultural pest detection: A case study on whiteflies. Front Plant Sci 13:813050. 10.3389/fpls.2022.813050 PMC9523729 36186035

[CR31] Kargar A, Zorbas D, Tedesco S et al (2024) Detecting halyomorpha halys using a low-power edge-based monitoring system. Comput Electron Agric 221:108935. 10.1016/j.compag.2024.108935

[CR32] Khairunniza-Bejo S, Ibrahim MF, Hanafi M, et al (2024) Automatic paddy planthopper detection and counting using faster r-cnn. Agriculture 14(9). 10.3390/agriculture14091567

[CR33] Le VL, Beurton-Aimar M, Zemmari A et al (2020) Automated landmarking for insects morphometric analysis using deep neural networks. Eco Inform 60:101175. 10.1016/j.ecoinf.2020.101175

[CR34] Li H, Liang Y, Liu Y et al (2023) Development of an intelligent field investigation system for liriomyza using seresnet-liriomyza for accurate identification. Comput Electron Agric 214:108276. 10.1016/j.compag.2023.108276

[CR35] Li W, Zheng T, Yang Z et al (2021) Classification and detection of insects from field images using deep learning for smart pest management: A systematic review. Eco Inform 66:101460. 10.1016/j.ecoinf.2021.101460

[CR36] Liu W, Wu G, Ren F et al (2020) Dff-resnet: An insect pest recognition model based on residual networks. Big Data Min Analyt 3(4):300–310. 10.26599/BDMA.2020.9020021

[CR37] Liu W, Wu G, Ren F (2021) Deep multibranch fusion residual network for insect pest recognition. IEEE Trans Cogn Developm Syst 13(3):705–716. 10.1109/TCDS.2020.2993060

[CR38] Maurício J, Domingues I, Bernardino J (2023) Comparing vision transformers and convolutional neural networks for image classification: A literature review. Appl Sci 13(9):5521. 10.3390/app13095521

[CR39] Molina-Rotger M, Morán A, Miranda MA et al (2023) Remote fruit fly detection using computer vision and machine learning-based electronic trap. Front Plant Sci 14–2023. 10.3389/fpls.2023.1241576 PMC10595146 37881610

[CR40] Nanni L, Maguolo G, Pancino F (2020) Insect pest image detection and recognition based on bio-inspired methods. Eco Inform 57:101089. 10.1016/j.ecoinf.2020.101089

[CR41] Page MJ, McKenzie JE, Bossuyt PM, et al (2021) The prisma 2020 statement: an updated guideline for reporting systematic reviews. bmj 372. 10.1136/bmj.n71 PMC8005924 33782057

[CR42] Peng Y, Wang Y (2022) Cnn and transformer framework for insect pest classification. Eco Inform 72:101846. 10.1016/j.ecoinf.2022.101846

[CR43] Proença MC, Rebelo MT, Valent R et al (2025) Identification of green leafhoppers (cicadellidae) in vineyards through an automatic image acquisition system from yellow sticky traps associated with deep-learning. Ciência Téc Vitiv 40(1):1–9. 10.1051/ctv/ctv2025400101

[CR44] Rustia DJA, Chao JJ, Chiu LY et al (2021) Automatic greenhouse insect pest detection and recognition based on a cascaded deep learning classification method. J Appl Entomol 145(3):206–222. 10.1111/jen.12834

[CR45] Rustia DJA, Lee WC, Lu CY et al (2023) Edge-based wireless imaging system for continuous monitoring of insect pests in a remote outdoor mango orchard. Comput Electron Agric 211:108019. 10.1016/j.compag.2023.108019

[CR46] Saud Yonbawi SMMSultan Alahmari (2023) Modified metaheuristics with transfer learning based insect pest classification for agricultural crops. Comput Syst Sci Eng 46(3):3847–3864. 10.32604/csse.2023.036552

[CR47] Shah S, Tembhurne J (2023) Object detection using convolutional neural networks and transformer-based models: a review. J Electric Syst Inf Technol 10(1):54. 10.1186/s43067-023-00123-z

[CR48] She J, Zhan W, Hong S et al (2022) A method for automatic real-time detection and counting of fruit fly pests in orchards by trap bottles via convolutional neural network with attention mechanism added. Eco Inform 70:101690. 10.1016/j.ecoinf.2022.101690

[CR49] Shi C, Zhang C, Zhang B et al (2025) Introduction risk assessment for quarantine pests by environmental monitoring, object detection and monte carlo simulation. Comput Electron Agric 233:110132. 10.1016/j.compag.2025.110132

[CR50] de Souza GS, Corrêa Vargas C, Hamerski JC (2025) Automatic detection and counting of tuta absoluta insect in trap images. Revista de Informática Teórica e Aplicada 32(1):47–53. 10.22456/2175-2745.143522

[CR51] Sun G, Liu S, Luo H et al (2022) Intelligent monitoring system of migratory pests based on searchlight trap and machine vision. Front Plant Sci 13–2022. 10.3389/fpls.2022.897739 PMC9251472 35795344

[CR52] Sun W, Li Y, Feng H et al (2024) Lightweight and accurate aphid detection model based on an improved deep-learning network. Eco Inform 83:102794. 10.1016/j.ecoinf.2024.102794

[CR53] Vilar-Andreu M, García L, Garcia-Sanchez AJ et al (2024) Enhancing precision agriculture pest control: A generalized deep learning approach with yolov8-based insect detection. IEEE Access 12:84420–84434. 10.1109/ACCESS.2024.3413979

[CR54] Wang D, Wang Y, Li M et al (2021) Using an improved yolov4 deep learning network for accurate detection of whitefly and thrips on sticky trap images. Trans ASABE 64(3):919–927. 10.13031/trans.14394

[CR55] Wang F, Liu L, Dong S et al (2022) Asp-det: Toward appearance-similar light-trap agricultural pest detection and recognition. Front Plant Sci 13:864045. 10.3389/fpls.2022.864045 PMC9297926 35874026

[CR56] Wang N, Fu S, Rao Q et al (2025) Insect-yolo: A new method of crop insect detection. Comput Electron Agric 232:110085. 10.1016/j.compag.2025.110085

[CR57] Wang QJ, Zhang SY, Dong SF et al (2020) Pest24: A large-scale very small object data set of agricultural pests for multi-target detection. Comput Electron Agric 175:105585. 10.1016/j.compag.2020.105585

[CR58] Wang S, Chen D, Xiang J, et al (2024) A deep-learning-based detection method for small target tomato pests in insect traps. Agronomy 14(12). 10.3390/agronomy14122887

[CR59] Wei M, Zhan W (2024) Yolo_mrc: A fast and lightweight model for real-time detection and individual counting of tephritidae pests. Eco Inform 79:102445. 10.1016/j.ecoinf.2023.102445

[CR60] Wu Q, Zeng J, Wu K (2022) Research and application of crop pest monitoring and early warning technology in china. Front Agricult Sci Eng 9(1):19–36. 10.15302/J-FASE-2021411

[CR61] Xiang Q, Huang X, Huang Z, et al (2023) Yolo-pest: An insect pest object detection algorithm via cac3 module. Sensors 23(6). 10.3390/s23063221 PMC10059078 36991930

[CR62] Yao Q, Feng J, Tang J et al (2020) Development of an automatic monitoring system for rice light-trap pests based on machine vision. J Integr Agric 19(10):2500–2513. 10.1016/S2095-3119(20)63168-9

[CR63] Zhang W, Huang H, Sun Y et al (2022) Agripest-yolo: A rapid light-trap agricultural pest detection method based on deep learning. Front Plant Sci 13:1079384. 10.3389/fpls.2022.1079384 PMC9800973 36589124

[CR64] Zhang W, Wang Y, Shen G et al (2025) Detection of bemisia tabaci based on swinir super-resolution reconstruction and semantic-sam model. Comput Electron Agric 237:110667. 10.1016/j.compag.2025.110667

[CR65] Zhang Z, Rong J, Qi Z et al (2024) A multi-species pest recognition and counting method based on a density map in the greenhouse. Comput Electron Agric 217:108554. 10.1016/j.compag.2023.108554

[CR66] Zhou C, Lee WS, Zhang S et al (2024) A smartphone application for site-specific pest management based on deep learning and spatial interpolation. Comput Electron Agric 218:108726. 10.1016/j.compag.2024.108726

