# Supplementary material for: Fine-Grained Recognition of Insect Pests from Digital Images: A Survey
Source: Neotrop Entomol. 2026 May 4;55(1):48. doi: 10.1007/s13744-026-01385-8 (PMC13139295; doi:10.1007/s13744-026-01385-8)
Supplement: Supplementary file 1 — (pdf 212 KB) [file 13744_2026_1385_MOESM1_ESM.pdf]

Supplementary Materials for:

Fine-grained Recognition of Insect Pests from Digital Images: A Survey

Telmo De Cesaro Júnior, et al.

March 2026

Supplementary Table S1: Detailed Analysis of Reviewed Studies

Table 1: Systematic literature review data (2020-2025).

Ref.	Country	Crop	Species	Trap	CV/DL Methods	IPM Application Context
Wang et al. (2024)	China	Tomato	Whitefly, *Macrolophus*	Sticky trap	YOLOv7-tiny (Transformer elements)	No
Ciampi et al. (2023)	Italy	Vegetable crops in greenhouses	Whitefly (*Bemisia tabaci*, *Trialeurodes vaporariorum*)	Yellow sticky trap	Deep Learning. Task: Abundance estimation (counting) and localization. Modular pipeline: Counting by detection (Faster R-CNN, FCOS), by regression for density maps (CSRNet, FCRN), or for segmentation maps (U-Net)	No climate data cross-referencing
Zhang et al. (2022)	China	Field crops	24 classes of typical agricultural pests (e.g., *Heliothis armigera*, *Sesamia inferens*)	Luminous	Deep Learning (improved YOLOv5s). Task: Object detection (derived counting). Customizations: CLA, GSPPF, Soft-NMS	No climate data cross-referencing
She et al. (2022)	China	Fruit orchards, melons and vegetables	Melon fruit fly (*Bactrocera cucurbitae*)	Pheromone trap bottles; e-trap	Deep Learning (YOLOv5). Task: Object detection and counting. Customizations: Anchor redesign, attention mechanisms (ECA), consecutive frame processing, Hough circle detection	No climate data cross-referencing
Huang et al. (2021)	China	Citrus orchards	Fruit flies (*Bactrocera minax*, *B. dorsalis*, *B. scutellata*)	Motorized e-trap with Yellow sticky trap	Deep Learning (CNN). Task: Object detection, classification and counting. Model: MAMPNet (Multi-Attention and Multi-Part CNN) with auxiliary losses (LSP, LCH) and sampling difference detection algorithm	Integrated: meteorological data collected (temperature and humidity), but the data cross-referencing is not explicit
Zhang et al. (2024)	China	Greenhouses tomato crops	Whiteflies (*Trialeurodes vaporariorum*) and fruit flies (*Drosophila melanogaster*)	Yellow sticky trap	Deep Learning (density maps). Task: Counting and multi-species recognition (pixel-based regression). Model: MS-DM (VGG19 backbone, FPN, ASEA, ConF - ASPP + CBAM)	No climate data cross-referencing
Checola et al. (2024)	Italy	Grapevines	Golden flavescence leafhopper (*Scaphoideus titanus*), grape moth (*Lobesia botrana*), mosaic leafhopper (*Orientus ishidae*)	Yellow sticky trap; e-trap and scanning	Deep Learning. Task: Object detection. Models: YOLOv8, Faster R-CNN (ResNet-50 backbone, FPN)	No climate data cross-referencing
Bai et al. (2024)	China	Cotton crops	Multiple species (Bee, Black stink bug, Pink bollworm, Lacewing, Fly, Ladybug, Thrips, White-backed planthopper, Whitefly)	Yellow sticky trap	Deep Learning. Task: Counting, localization and classification (point-based method). Model: MS-P2P (YOLOv7-tiny + LAHead)	No climate data cross-referencing
Zhou et al. (2024)	USA	Strawberry cultivation	Two-spotted spider mite (TSSM), Two-spotted spider mite egg, Predatory mite (PM), Predatory mite egg	No (direct images on the plant); acquisition via smartphone	Deep Learning (YOLOv7 tiny). Task: Object detection for counting. Spatial interpolation (IDW) for distribution mapping	Contextual: focused on managing specific local pests. No climate data cross-referencing

Table 1: Systematic literature review data (2020-2025).

**Ref.**	**Country**	**Crop**	**Species**	**Trap**	**CV/DL Methods**	**IPM Application Context**
Wang et al. (2022)	China	Field environments	Typical agricultural pests (24 categories with similarity in appearance/scale)	Luminous	Deep Learning. Task: Object detection (ASPD). Model: ASP-Det (PSA, Non-Local Modules, SCC, center-ness calibration)	Contextual: intended for an efficient pest control system. No climate data cross-referencing
Le et al. (2020)	France	Agricultural fields (agroecology context)	*Poecilus cupreus* (carabid beetle, beneficial predator)	No	Deep Learning (CNN). Task: Prediction of landmarks for morphometric analysis. Model: EB-Net (Elementary Block Network)	Focuses on beneficial insects. No climate data cross-referencing
de Souza et al. (2025)	Brazil	Tomato production	*Tuta absoluta* (tomato leafminer)	Pheromone	Deep Learning. Task: Object detection and counting. Models: YOLOv3 and TinyYOLOv3	Contextual: integrates AI for monitoring and alerts. No climate data cross-referencing
Rustia et al. (2021)	Taiwan	Tomato and Lisianthus seedlings (greenhouses)	Flies, mosquitoes, thrips and whiteflies	Adhesive; e-trap	Deep Learning (CNNs). Task: Object detection and classification (multi-class). Cascaded approach (object detector, insect/non-insect classifier, multi-class classifier)	No climate data cross-referencing
Guo et al. (2023)	China	Vegetable field	*Plutella xylostella*, *Bemisia tabaci*, *Frankliniella occidentalis*, *Phyllotreta striolata*, *Bactrocera cucurbitae*	Yellow sticky trap; e-trap	Deep Learning (YOLO-SIP). Task: Object detection and counting (abundance estimation)	Contextual: basis for pest management. No climate data cross-referencing
Khairunniza-Bejo et al. (2024)	Malaysia	Rice fields	Brown planthopper (BPH), White-backed planthopper (WBPH), Zigzag leafhopper (ZIGZAG), Green leafhopper (GLH), Benign insects	Luminous adhesive; e-trap	Deep Learning (Faster R-CNN VGG-16). Task: Object detection, classification and counting	Contextual: crucial for optimizing interventions. No climate data cross-referencing
Hong et al. (2021)	Soth Korea	Forest plantations (Black pine)	Black pine scale: *Matsucoccus thunbergianae*	Pheromone	Deep Learning. Task: Object detection and counting. Models: Faster R-CNN Resnet 101	No climate data cross-referencing
Peng and Wang (2022)	China	Common agricultural pests (various crops)	102 species (IP102), 40 categories (D0), 10 species (Li's dataset)	No	Deep Learning (hybrid CNN and Transformer). Task: Classification. Backbone: ResNet50. Attention-based classification head	No climate data cross-referencing
Dong et al. (2021)	China	Arable crops (agricultural field)	26 pest classes (e.g., *Spodoptera exigua*, *Heliothis armigera*)	E-trap	Deep Learning. Task: Object detection and classification. Model: CRA-Net (CRFPN, Adaptive Anchor Module). Backbone: ResNet-50	No climate data cross-referencing
Chen et al. (2025b)	China	Agricultural production (general monitoring)	24 pest classes (e.g., *Gryllotalpa spp*, *Mythimna separata*, *Spodoptera exigua*)	Luminous; e-trap	Deep Learning. Task: Multi-scale object detection and recognition/classification (derived counting). Model: DAMI-YOLOv8l (DMC, ASF-P2, MPDinner-IoU)	No climate data cross-referencing

Table 1: Systematic literature review data (2020-2025).

Ref.	Country	Crop	Species	Trap	CV/DL Methods	IPM Application Context
Ibrahim et al. (2023)	Malaysia	Rice crops	Rice planthoppers (Brown Planthopper, Green Leafhopper, White-backed Planthopper, Zigzag Leafhopper)	Yellow sticky trap and luminous	Deep Learning (CNNs). Task: Multi-species classification (implicit counting). Model: ResNet-50	No climate data cross-referencing
Freitas et al. (2022)	Brazil	Fruit orchards	*Ceratitis capitata* (Mediterranean fruit fly) and *Grapholita molesta* (oriental fruit moth)	Pheromone; e-trap	Deep Learning (CNN). Task: Identification, classification and counting. Model: QResNet (ResNet18)	Contextual: population monitoring. No climate data cross-referencing
Liu et al. (2021)	China / Japan	Crop fields (common)	102 species (IP102), D0, Li's dataset	No	Deep Learning (hybrid CNN and ViT). Task: Recognition and classification. Model: DMF-ResNet (SFR module, attention-based classification head)	Contextual: aimed at evaluating agricultural yield. No climate data cross-referencing
Kargar et al. (2024)	Italy	Pear orchards	Brown marmorated stink bug (*Halyomorpha halys*)	Yellow sticky trap; e-trap	Deep Learning, Machine Learning. Task: Detection and classification. Model: CNN with Depthwise Separable Convolution (DwSConv)	No climate data cross-referencing
De Castro Pereira et al. (2022)	Brazil	Soybean crops (in laboratory)	Whitefly (*Bemisia tabaci*) and its five developmental stages	No (direct images on the plant)	Deep Learning (CNN). Task: Detection and classification of developmental stages (counting). Model: Enhanced YOLOv4 (YOLOv4 cascade, data augmentation by cropping, image mosaic)	No climate data cross-referencing
Zhang et al. (2025)	China	Tobacco cultivation	Tobacco whitefly (*Bemisia tabaci*): eggs, nymphs (1st, 2nd, 3rd, 4th instar), adults	No (direct detection on the plant with microscope)	Deep Learning (ViT). Task: Target identification, detection and classification of stages. Model: SwinIR (super-resolution) and Semantic-SAM (semantic segmentation) with Feret Diameter	Contextual: prediction and control. No climate data cross-referencing
Gao et al. (2024)	China	Sugar beet fields	Aphids	Yellow water tray	Deep Learning (hybrid CNN). Task: Automatic counting (object detection and density map estimation). Model: Improved YOLOv5 + CSRNet (alternates between detection and density)	Contextual: early warning. No climate data cross-referencing
Chou et al. (2023)	Taiwan	Asparagus cultivation (in greenhouse)	Whiteflies and thrips	Yellow sticky trap; e-trap (AIoT)	Deep Learning. Task: Object detection for counting. Model: YOLOv5 with x6 backbone	Integrated: optimization of crop management. Cross-references with climate and environmental data (temperature, relative humidity, soil temperature, etc.)
Yao et al. (2020)	China	Rice fields	Large pests (*Sesamia inferens*, *Chilo suppressalis*, *Cnaphalocrocis medinalis*), Small pests (*Sogatella furcifera*)	Luminous; e-trap	Deep Learning (CNNs). Task: Detection and individual identification, counting. Models: ResNet-50 (large), ResNet-18 (small)	No climate data cross-referencing

Table 1: Systematic literature review data (2020-2025).

**Ref.**	**Country**	**Crop**	**Species**	**Trap**	**CV/DL Methods**	**IPM Application Context**
Li et al. (2023)	China	Agricultural fields (various vegetable crops)	Leaf miners: *Liriomyza* (*L. sativae*, *L. huidobrensis*, *L. trifolii*, *L. chinensis*, *L. bryoniae*)	Yellow sticky trap and light (acquisition by smartphone)	Deep Learning (CNN). Task: Classification for identification. Model: SeResNet-Liriomyza (SFPN)	Contextual: real-time monitoring. Does not integrate with climate data
Liu et al. (2020)	Japan	Crops in general	Insect pests in general	No	Deep Learning (CNN). Task: Recognition and localization. Model: DFF-ResNet	Does not integrate with climate data
Hechen et al. (2024)	China	Citrus and crops in general	Citrus mites (6 types), 102 species of IP102	Not specified (existing datasets)	Deep Learning (ViT). Task: Classification. Model: DWViT-ES (ESA, DW, SSA)	Does not integrate with climate data
Rustia et al. (2023)	Taiwan	Mango orchard	*Cicadellidae* (leafhoppers), *Diptera* (flies), *Thrips* (thrips). Main pests: Thrips, mango leafhopper, oriental fruit fly	Adhesive; e-trap (network)	Deep Learning (Tiny YOLO v3 + lightweight CNNs). Task: Object detection and classification (hierarchical). Tree-based classifier	Integrated: real-time data generation to aid decision-making with climate data (temperature, precipitation, light, etc.)
Gonçalves et al. (2022)	Portugal	Viticulture (vineyards/grapes)	Green leafhopper (*Empoasca vitis*), Grapevine moth (*Lobesia botrana*), Tomato leafminer (*Tuta absoluta*), *Idaea degeneraria*, *Theresimima ampelophaga*	Yellow and delta sticky traps (conventional, photos by smartphone)	Deep Learning (CNNs). Task: Object detection for identification and counting. Models: SSD ResNet50, CenterNet ResNet50, SSD MobileNet V2, EfficientDet-D0, Faster R-CNN ResNet101	Does not integrate with climate data
Čirjak et al. (2023a)	Croatia	Apple orchards	Apple moth: Cydia pomonella (and other insects/objects)	Pheromone: e-trap	Deep Neural Network: EfficientDet-4 (ED4) for object detection	Contextual: (remote population monitoring for early detection of damage and temperature/humidity data)
Vilar-Andreu et al. (2024)	Spain	Generalist perspective (any crop)	Any type of insect (grouped as insect)	No (focus on generalized detection)	Deep Learning (CNN). Task: Object detection (generalized presence detection). Model: YOLOv8 (nano, small, medium, large, extra large)	Does not integrate with climate data
Li et al. (2021)	China	Agricultural greenhouses	Whiteflies and thrips	Yellow sticky trap; e-trap	Deep Learning (Faster R-CNN). Task: Object detection and population estimation. Model: TPest-RCNN (VGG16, RoIAlign, anchor optimization for small objects)	Does not integrate with climate data
Amarathunga et al. (2022)	Australia	Horticulture (flower harvesting)	Thrips: Western flower thrips (*Frankliniella occidentalis*) and Plague thrips (*Thrips imaginis*)	No (specimens collected and mounted on slides)	Deep Learning (ViT). Task: Fine grain classification. Model: Domain Knowledge-Driven Stacked Model	Does not integrate with climate data
Proença et al. (2025)	Portugal	Vineyards	Green leafhoppers (*H. solani/ pteridis* and *J. lybica*)	Yellow sticky trap (iSCOUT® COLOR TRAP); e-trap	Deep Learning (CNN). Task: Classification and counting. Model: YOLOv7 (optimization for lighting/orientation, tiling)	Does not integrate with climate data

Table 1: Systematic literature review data (2020-2025).

**Ref.**	**Country**	**Crop**	**Species**	**Trap**	**CV/DL Methods**	**IPM Application Context**
De Cesaro Júnior et al. (2022)	Brazil	Wheat crops	Aphids and parasitoids	Moericke type tray (images digitized in the laboratory)	Deep Learning (CNNs). Task: Object detection. Model: Mask R-CNN	Does not integrate with climate data
Nanni et al. (2020)	Italy	Tea and other crops	10 species (*Locusta migratoria*, *Parasa lepida*, Gypsy moth larva, *Empoasca flavescens*, *Spodoptera exigua*, *Chrysocus chinensis*, *Laspeyresia pomonella* larva, *Spodoptera exigua* larva, *Atractomorpha sinensis*, *Laspeyresia pomonella*)	No	CNN and saliency methods. Task: Classification. Model: Ensemble of classifiers	Does not integrate with climate data
Wang et al. (2025)	China	Grain crops (rice, corn, wheat)	7 species (*Loxostege sticticalis*, *Pyrausta nubilalis*, *Sesamia inferens*, *Chilo suppressalis*, *Cnaphalocrocis medinalis*, *Spodoptera frugiperda*, rice planthopper)	Luminous; e-trap	Deep Learning (CNNs). Task: Object detection, recognition and counting. Model: YOLOv8m with CBAM	Does not integrate with climate data
Sun et al. (2022)	China	Rice crops	Migratory rice pests (*Cnaphalocrocis medinalis*, *Sogatella furcifera*, *Nilaparvata lugens*)	Searchlight trap; e-trap	Deep Learning (CNN). Task: Object detection and counting. Model: YOLO-MPNet (YOLOv4 + SENet + SPP) + OSW	Does not integrate with climate data
Shi et al. (2025)	China	Non-agricultural (cargo holds)	Quarantine pests (Bruchid beetle, Hemiptera long-horned beetle, Dermestid beetle, Cerambycidae)	Pheromone, luminous and Yellow sticky trap; e-trap	Deep Learning. Task: Object detection. Model: QPNet (DS + FE + SAI + ResNet50)	Integrated: for risk assessment of pest introduction. Integrates with environmental data (temperature, humidity, fumigant gas)
Sun et al. (2024)	China	Bamboo shoot fields	Winged bamboo aphid (*Takecallis taiwanus*)	Yellow sticky trap	Deep Learning. Task: Object detection. Model: SCA-YOLOv5s (ShuffleNetv2, CA, anchor box optimization)	Technical support for aphid control. Does not integrate with climate data
Saud Yonbawi (2023)	Saudi Arabi	Agricultural crops in general	Insect pests in General	No	Hybrid (Meta-heuristics with Transfer Learning + CNN). Task: Classification. Model: MMTL-IPCAC (CLAHE, NASNet, MGWO, XGBoost)	Does not integrate with climate data
Čirjak et al. (2023b)	Croatia	Apple orchards	Apple leaf miner (*Leucoptera malifoliella*), other insects, leaf mines (damage)	Pheromone (Delta type); e-trap	Artificial Neural Networks (ANNs). Task: Object detection and classification (adult pest and damage). Model: EfficientDet Object identification (EfficientNet, BiFPN, separable convolutions)	Contextual: real-time monitoring for targeted control. Does not integrate with climate data

Table 1: Systematic literature review data (2020-2025).

Ref.	Country	Crop	Species	Trap	CV/DL Methods	IPM Application Context
Wang et al. (2020)	China	Agricultural crops (in general)	Insect pests in general (24 categories)	E-trap	Deep Learning (CNN). Task: Multi-target detection and counting. Models evaluated: YOLOv3 (best), Faster RCNN, RetinaNet, SSD	Does not integrate with climate data
Chen et al. (2025a)	China	Agricultural crops (corn, but general context)	24 types of agricultural pests (e.g., Armyworm, Cotton bollworm)	Multispectral e-trap	Deep Learning. Task: Object detection and counting (multi-class and dense). Model: Pest-PVT (PVTv2, SSA, FCOS, ATSS, DyHead)	Does not integrate with climate data
Hacinas et al. (2024)	Philippines	Cocoa plantations	Cacao Pod Borer (CPB)	Yellow sticky and pheromone (acquisition via smartphone)	Deep Learning (CNN). Task: Object detection. Model: YOLOv8 Large	Does not integrate with climate data
Molina-Rotger et al. (2023)	Span	Olive groves	Olive fruit fly (*Bactrocera oleae*), fruit flies (*B. minax*, *B. dorsalis*, *B. scutellata*)	Yellow sticky trap; e-trap	Machine Learning (Random Forest, SVM, Decision Tree) and Deep Learning (CNNs). Task: Detection and classification. Counting by difference analysis	Does not integrate with climate data
Geissmann et al. (2022)	Canada	Natural/ semi-natural environment	*Macropsis fuscula*, *Drosophila suzukii*, Drosophilids, *Anthonomus rubi*, *Psyllobora vigintimaculata*, *Coleoptera*, *Lasioglossum laevissimum*	Yellow sticky trap; e-trap	Deep Learning (CNNs). Task: Instance segmentation (Mask R-CNN) and tracking (Siamese network)	Not specified (focus on circadian activity). Does not integrate with climate data
Kalfas et al. (2023)	Belgium	Witloof chicory	Chicory leaf miners, woolly aphids, ichneumon wasps, grass flies	Yellow sticky trap	Deep Learning. Task: Object detection and classification. Model: YOLOv5	Contextual: early detection in hotspots. Does not integrate with climate data
Wang et al. (2021)	China	Vegetable greenhouses	Whiteflies and thrips	Yellow sticky trap; e-trap	Deep Learning (improved YOLOv4). Task: Object detection and population estimation. Customizations for small objects and robustness.	Contextual: Population monitoring. Does not integrate with climate data
Bollis et al. (2022)	Brazil	Citrus orchards	Citrus mites (6 types) and insect pests in general	No	Deep Learning. Task: Classification (with activation maps). Model: Attention-based MIL-Guided (EfficientNet-B0 backbone)	Does not integrate with climate data
Wei and Zhan (2024)	China	Melon, fruit and vegetable crops	Pumpkin fruit fly	Bottle trap (with pheromone); e-trap	Deep Learning. Task: Object detection and individual counting. Model: YOLO_MRC (improved YOLOv8n: Multicat, C2flite)	Contextual: to optimize pesticide use. Does not integrate with climate data
Xiang et al. (2023)	China	Agricultural crops in general	Insect pests in general (102 categories IP102, 28 types Teddy Cup)	No	Deep Learning (CNN and ViT). Task: Object detection. Model: Yolo-Pest (improved YOLOv5s: CAC3 module, SE, ConvNeXt)	Contextual: relevant for crop loss investigation. Does not integrate with climate data
Huang et al. (2025)	China	Monoculture crops (agricultural field)	Whiteflies (WF), flea beetles (SFB) and Syrphidae (SP), *Macrolophus* (MR) and *Nesidiocoris* (NC)	Yellow sticky trap; e-trap	Deep Learning. Task: Object detection. Model: Improved YOLOv10n (dual assignment, CIB, subsampling separation, data augmentation)	Contextual: provides crucial technical support for IPM. Does not integrate with climate data

Table 1: Systematic literature review data (2020-2025).

Ref.	Country	Crop	Species	Trap	CV/DL Methods	IPM Application Context

Supplementary Table S2: Group A - Fine-Grained Visual Classification (FGVC) Studies

Table 2: Detailed imaging resources, deep learning approaches, and performance metrics for FGVC studies.

Reference	Acquisition Source	Model / Method	Metric / Performance
Amarathunga et al. (2022)	Portable digital microscope	ViT, segmentation and attention	Accuracy: 97.8%
De Castro Pereira et al. (2022)	Panasonic DMC-LZ10 camera	YOLOv4 (CNN) + custom modules	F1-score: 0.87
Zhang et al. (2025)	Microscopy, super-resolution	SwinIR (ViT)	Accuracy: $<$ 90%
Bollis et al. (2022)	IP102/CBP dataset	Two-WAM (CNN) and EfficientNet-B0	Accuracy: 92.4%
Hechen et al. (2024)	IP102 dataset	DMF-ResNet, attention (ViT)	Accuracy: 76.0%
Liu et al. (2020)	IP102 dataset	SFR (CNN)	Acc: 55.4%, F1: 54%
Li et al. (2023)	Smartphone + magnifying lens	SeResNet-Liriomyza (CNN)	Accuracy: 99.9%
Peng and Wang (2022)	IP102/DO/Li datasets	Attention head (CNN/ViT)	Accuracy: 74.9%
Liu et al. (2021)	IP102/DO/Li datasets	SFR + DMF-ResNet + attention	Accuracy: 59.2%
Hong et al. (2021)	High-resolution camera	Faster R-CNN (CNN)	Accuracy: 97.9%
Ibrahim et al. (2023)	Industrial camera (MV-CA060)	ResNet-50 and VGG (CNN)	Accuracy: 97.3%
Zhou et al. (2024)	Smartphone, 25X macro lens	YOLOv7-tiny	Accuracy: 76.7%
Nanni et al. (2020)	IP102 dataset, SLR camera	Saliency methods (CNN)	Accuracy: 61.9%
Xiang et al. (2023)	IP102/Teddy CUP datasets	Hybrid approach (CNN/ViT)	mAP@0.5: 57.1%
De Cesaro Júnior et al. (2022)	1200 dpi scanner	Mask R-CNN (CNN)	mAP: 68.3%
Kalfas et al. (2023)	High-resolution camera	YOLOv5 (CNN)	mAP: 0.76
Wang et al. (2022)	PestNet-AS dataset, e-trap	ASP-Det (CNN)	AP: 45.0%
Wang et al. (2025)	E-trap	Insect-YOLO + CBAM (CNN)	mAP@50: 93.8%
Chen et al. (2025a)	Pest24 dataset, e-trap	Pest-PVT, multi-scale attention (ViT)	mAP: 77.2%
Chen et al. (2025b)	LP24 dataset, e-trap	DAMI-YOLOv8l (CNN)	mAP@50: 78.2%
Dong et al. (2021)	LMPD2020 dataset, e-trap	CRA-Net, adaptive anchor (CNN)	mAP@50: 74.8%
Le et al. (2020)	Digital camera, trinocular	EB-Net (CNN)	Landmarks extraction
Zhang et al. (2024)	Smartphone camera	MS-DM, ConF module	Not informed
Wang et al. (2024)	Digital camera	YOLOv7-tiny + CIE block	mAP: 90.4%
Bai et al. (2024)	Smartphone camera	MS-P2P (YOLOv7), LAHead	mAP@0.5: 86.4%
Zhang et al. (2022)	Pest24 dataset	AgriPest-YOLO (YOLOv5s)	mAP: 90.4%
Sun et al. (2024)	Smartphone camera	SCA-YOLOv5s, CA attention	mAP@0.5: 92.2%

Supplementary Table S3: Group B - Generic Detection and Classification Studies

Table 3: Detailed imaging resources and performance metrics for generic studies.

Reference	Acquisition Source	Model / Method	Metric / Performance
Saud Yonbawi (2023)	Not informed	CNN: MMTL-IPCAC	Accuracy: 98.7%
Vilar-Andreu et al. (2024)	Not informed	CNN: YOLOv8	mAP@50: 97.0%
Wang et al. (2021)	YST - e-trap	YOLOv4	mAP: 92.7%
Li et al. (2021)	YST - e-trap	CNN: TPest-RCNN	AP: 95.0%
Gonçalves et al. (2022)	YST - Smartphone	SSD ResNet50	Accuracy: 82.0%
Ciampi et al. (2023)	YST - Digital Camera	CNN: Modular Pipeline	MARE: 9.3%
Gao et al. (2024)	YST - Digital Camera	CNN: YOLOv5	AP: 65.0%
Hacinas et al. (2024)	YST - Smartphone	YOLOv8l	F1-Score: 0.88
de Souza et al. (2025)	Pheromones - Smartphone	YOLOv3	mAP: 95.3%
Wang et al. (2020)	E-trap	CNN: YOLOv3	mAP: 63.5%

Supplementary Table S4: E-trap maturation regarding deployment scale and validation period

Table 4: E-trap approaches: deployment scale and validation period of real-time monitoring systems.

Reference	e-Trap Units	Validation Time
Rustia et al. (2023)	8 units	2 years
Chou et al. (2023)	3 units	7 days
Guo et al. (2023)	20 units	3 months
Rustia et al. (2021)	Multiple units (3 sites)	6 months
Čirjak et al. (2023b)	2 units	Not informed
Proença et al. (2025)	2 commercial units	5 months
Huang et al. (2025)	1 unit	5 months
Geissmann et al. (2022)	10 units	4 months
Kargar et al. (2024)	1 unit	Not informed
Huang et al. (2021)	1 unit	Not informed
Molina-Rotger et al. (2023)	2 units	5 months
Čirjak et al. (2023a)	10 units	4 months
Freitas et al. (2022)	Multiple units	Not informed
She et al. (2022)	1 unit	Not informed
Sun et al. (2022)	1 unit	Not informed
Yao et al. (2020)	4 units	6 months
Khairunniza-Bejo et al. (2024)	1 unit	Not informed
Wei and Zhan (2024)	1 unit	Not informed
Checola et al. (2024)	Not informed	Not informed
Shi et al. (2025)	Not informed	Not informed

Supplementary Table S5: High-resolution and microscopy resources for Fine-Grained Visual Classification (FGVC)

Table 5: Detailed microscopy and industrial camera resources used in FGVC studies.

Reference	Technology / Hardware	Description / Application
**Microscopes and Macro Lenses**		
Amarathunga et al. (2022)	Portable digital microscope (USB)	Image capture of microscopic thrips.
Li et al. (2023)	Magnifying lens for smartphone	Identification of *Liriomyza* spp. with similar morphological traits.
Le et al. (2020)	Trinocular magnifier	Morphometric analysis of beetles.
Zhou et al. (2024)	25x macro lens for smartphone	Detection of small mites.
Zhang et al. (2025)	Microscope + digital camera	Capture of *Bemisia tabaci* and its developmental stages.
**High-Resolution Industrial Cameras**		
Ibrahim et al. (2023)	Industrial camera (MV-CA060-10GC)	Classification of similar planthoppers (1229 ppcm).
Kalfas et al. (2023)	Fujifilm 24 MP digital camera	High-resolution capture (6240 x 4160 px) for YOLOv5 training.

Supplementary Table S6: Performance comparison (mAP@0.5) across different datasets and models

Table 6: Comparative performance of AI models using public and private datasets.

**Reference**	**Model / Method**	**Dataset**	**mAP@0.5**
Chen et al. (2025a)	Pest-PVT	Pest24	77.2%
Chen et al. (2025b)	DAMI-YOLOv8l	LP24	78.2%
Shi et al. (2025)	QPNet	Pest24	71.5%
Zhang et al. (2022)	AgriPest-YOLO	Pest24	71.3%
Wang et al. (2025)	Insect-YOLO	PASCAL VOC2012	63.7%
Wang et al. (2020)	YOLOv3	Pest24	63.5%
Wei and Zhan (2024)	YOLO-MRC	Pest24-640	59.2%
Xiang et al. (2023)	Yolo-Pest	IP102 / Teddy Cup	57.1% / 91.9%
Khairunniza-Bejo et al. (2024)	Faster R-CNN (VGG-16)	Private	97.7%
She et al. (2022)	YOLOv5s-ECA	Private (Video)	95.9%
Wang et al. (2024)	Improved YOLOv7-tiny	Private	90.4%
Huang et al. (2025)	YOLO-YSTs	Private (YST)	86.8%

References

Amarathunga DC, Ratnayake MN, Grundy J, et al (2022) Fine-grained image classification of microscopic insect pest species: Western flower thrips and plague thrips. Computers and Electronics in Agriculture 203:107462. https://doi.org/10.1016/j.compag.2022.107462

Bai M, Chen T, Yuan J, et al (2024) A point-based method for identification and counting of tiny object insects in cotton fields. Computers and Electronics in Agriculture 227:109648. https://doi.org/10.1016/j.compag.2024.109648

Bollis E, Maia H, Pedrini H, et al (2022) Weakly supervised attention-based models using activation maps for citrus mite and insect pest classification. Computers and Electronics in Agriculture 195:106839. https://doi.org/10.1016/j.compag.2022.106839

Checola G, Bertoni L, Angeli S (2024) A novel dataset and deep learning object detection benchmark for grapevine pest surveillance. Frontiers in Plant Science 15:1485216. https://doi.org/10.3389/fpls.2024.1485216

Chen H, Wen C, Zhang L, et al (2025a) Pest-pvt: A model for multi-class and dense pest detection and counting in field-scale environments. Computers and Electronics in Agriculture 230:109864. https://doi.org/10.1016/j.compag.2024.109864

Chen X, Yang X, Hu H, et al (2025b) Dami-yolov8l: A multi-scale detection framework for light-trapping insect pest monitoring. Ecological Informatics 86:103067. https://doi.org/10.1016/j.ecoinf.2025.103067

Chou CY, Chang SC, Zhong ZP, et al (2023) Development of aiot system for facility asparagus cultivation. Computers and Electronics in Agriculture 206:107665. https://doi.org/10.1016/j.compag.2023.107665

Ciampi L, Zeni V, Incrocci L, et al (2023) A deep learning-based pipeline for whitefly pest abundance estimation on chromotropic sticky traps. Ecological Informatics 78:102384. https://doi.org/10.1016/j.ecoinf.2023.102384

De Castro Pereira R, Hirose E, Ferreira de Carvalho OL, et al (2022) Detection and classification of whiteflies and development stages on soybean leaves images using an improved deep learning strategy. Computers and Electronics in Agriculture 199:107132. https://doi.org/10.1016/j.compag.2022.107132

De Cesaro Júnior T, Rieder R, Di Domênico JR, et al (2022) Insectcv: A system for insect detection in the lab from trap images. Ecological Informatics 67:101516. https://doi.org/10.1016/j.ecoinf.2021.101516

Dong S, Wang R, Liu K, et al (2021) Cra-net: A channel recalibration feature pyramid network for detecting small pests. Computers and Electronics in Agriculture 191:106518. https://doi.org/10.1016/j.compag.2021.106518

Freitas L, Martins V, de Aguiar M, et al (2022) Deep learning embedded into smart traps for fruit insect pests detection. ACM Trans Intell Syst Technol 14(1). https://doi.org/10.1145/3552435

Gao X, Xue W, Lennox C, et al (2024) Developing a hybrid convolutional neural network for automatic aphid counting in sugar beet fields. Computers and Electronics in Agriculture 220:108910. https://doi.org/10.1016/j.compag.2024.108910

Geissmann Q, Abram PK, Wu D, et al (2022) Sticky pi is a high-frequency smart trap that enables the study of insect circadian activity under natural conditions. PLOS Biology 20(7):1–26. https://doi.org/10.1371/journal.pbio.3001689

Gonçalves J, Silva E, Faria P, et al (2022) Edge-compatible deep learning models for detection of pest outbreaks in viticulture. Agronomy 12(12). https://doi.org/10.3390/agronomy12123052

Guo Q, Wang C, Xiao D, et al (2023) Automatic monitoring of flying vegetable insect pests using an rgb camera and yolo-sip detector. Precision Agriculture 24(2):436–457. https://doi.org/10.1007/s11119-022-09952-w

Hacinas EAS, Querol LS, Santos KLT, et al (2024) Rapid automatic cacao pod borer detection using edge computing on low-end mobile devices. Agronomy 14(3). https://doi.org/10.3390/agronomy14030502

Hechen Z, Huang W, Yin L, et al (2024) Dilated-windows-based vision transformer with efficient-suppressive-self-attention for insect pests classification. Engineering Applications of Artificial Intelligence 127:107228. https://doi.org/10.1016/j.engappai.2023.107228

Hong SJ, Nam I, Kim SY, et al (2021) Automatic pest counting from pheromone trap images using deep learning object detectors for matsucoccus thunbergianae monitoring. Insects 12(4). https://doi.org/10.3390/insects12040342

Huang R, Yao T, Zhan C, et al (2021) A motor-driven and computer vision-based intelligent e-trap for monitoring citrus flies. Agriculture 11(5). https://doi.org/10.3390/agriculture11050460

Huang Y, Liu Z, Zhao H, et al (2025) Yolo-ysts: An improved yolov10n-based method for real-time field pest detection. Agronomy 15(3). https://doi.org/10.3390/agronomy15030575

Ibrahim MF, Khairunniza-Bejo S, Hanafi M, et al (2023) Deep cnn-based planthopper classification using a high-density image dataset. Agriculture 13(6). https://doi.org/10.3390/agriculture13061155

Čirjak D, Aleksi I, Lemic D, et al (2023a) Efficientdet-4 deep neural network-based remote monitoring of codling moth population for early damage detection in apple orchard. Agriculture 13(5). https://doi.org/10.3390/agriculture13050961

Čirjak D, Aleksi I, Miklečić I, et al (2023b) Monitoring system for leucoptera malifoliella (o. costa, 1836) and its damage based on artificial neural networks. Agriculture 13(1). https://doi.org/10.3390/agriculture13010067

Kalfas I, De Ketelaere B, Bunkens K, et al (2023) Towards automatic insect monitoring on witloof chicory fields using sticky plate image analysis. Ecological Informatics 75:102037. https://doi.org/10.1016/j.ecoinf.2023.102037

Kargar A, Zorbas D, Tedesco S, et al (2024) Detecting halyomorpha halys using a low-power edge-based monitoring system. Computers and Electronics in Agriculture 221:108935. https://doi.org/10.1016/j.compag.2024.108935

Khairunniza-Bejo S, Ibrahim MF, Hanafi M, et al (2024) Automatic paddy planthopper detection and counting using faster r-cnn. Agriculture 14(9). https://doi.org/10.3390/agriculture14091567

Le VL, Beurton-Aimar M, Zemmari A, et al (2020) Automated landmarking for insects morphometric analysis using deep neural networks. Ecological Informatics 60:101175. https://doi.org/10.1016/j.ecoinf.2020.101175

Li H, Liang Y, Liu Y, et al (2023) Development of an intelligent field investigation system for liriomyza using seresnet-liriomyza for accurate identification. Computers and Electronics in Agriculture 214:108276. https://doi.org/10.1016/j.compag.2023.108276

Li W, Wang D, Li M, et al (2021) Field detection of tiny pests from sticky trap images using deep learning in agricultural greenhouse. Computers and Electronics in Agriculture 183:106048. https://doi.org/10.1016/j.compag.2021.106048

Liu W, Wu G, Ren F, et al (2020) Dff-resnet: An insect pest recognition model based on residual networks. Big Data Mining and Analytics 3(4):300–310. https://doi.org/10.26599/BDMA.2020.9020021

Liu W, Wu G, Ren F (2021) Deep multibranch fusion residual network for insect pest recognition. IEEE Transactions on Cognitive and Developmental Systems 13(3):705–716. https://doi.org/10.1109/TCDS.2020.2993060

Molina-Rotger M, Morán A, Miranda MA, et al (2023) Remote fruit fly detection using computer vision and machine learning-based electronic trap. Frontiers in Plant Science Volume 14 - 2023. https://doi.org/10.3389/fpls.2023.1241576

Nanni L, Maguolo G, Pancino F (2020) Insect pest image detection and recognition based on bio-inspired methods. Ecological Informatics 57:101089. https://doi.org/10.1016/j.ecoinf.2020.101089

Peng Y, Wang Y (2022) Cnn and transformer framework for insect pest classification. Ecological Informatics 72:101846. https://doi.org/10.1016/j.ecoinf.2022.101846

Proença MdC, Rebelo, Maria Teresa, Valent, Riccardo, et al (2025) Identification of green leafhoppers (cicadellidae) in vineyards through an automatic image acquisition system from yellow sticky traps associated with deep-learning. Ciência Téc Vitiv 40(1):1–9. https://doi.org/10.1051/ctv/ctv2025400101

Rustia DJA, Chao JJ, Chiu LY, et al (2021) Automatic greenhouse insect pest detection and recognition based on a cascaded deep learning classification method. Journal of Applied Entomology 145(3):206–222. https://doi.org/10.1111/jen.12834

Rustia DJA, Lee WC, Lu CY, et al (2023) Edge-based wireless imaging system for continuous monitoring of insect pests in a remote outdoor mango orchard. Computers and Electronics in Agriculture 211:108019. https://doi.org/10.1016/j.compag.2023.108019

Saud Yonbawi SMMSultan Alahmari (2023) Modified metaheuristics with transfer learning based insect pest classification for agricultural crops. Computer Systems Science and Engineering 46(3):3847–3864. https://doi.org/10.32604/csse.2023.036552

She J, Zhan W, Hong S, et al (2022) A method for automatic real-time detection and counting of fruit fly pests in orchards by trap bottles via convolutional neural network with attention mechanism added. Ecological Informatics 70:101690. https://doi.org/10.1016/j.ecoinf.2022.101690

Shi C, Zhang C, Zhang B, et al (2025) Introduction risk assessment for quarantine pests by environmental monitoring, object detection and monte carlo simulation. Computers and Electronics in Agriculture 233:110132. https://doi.org/10.1016/j.compag.2025.110132

de Souza GS, Corrêa Vargas C, Hamerski JC (2025) Automatic detection and counting of tuta absoluta insect in trap images. Revista de Informática Teórica e Aplicada 32(1):47–53. https://doi.org/10.22456/2175-2745.143522

Sun G, Liu S, Luo H, et al (2022) Intelligent monitoring system of migratory pests based on searchlight trap and machine vision. Frontiers in Plant Science Volume 13 - 2022. https://doi.org/10.3389/fpls.2022.897739

Sun W, Li Y, Feng H, et al (2024) Lightweight and accurate aphid detection model based on an improved deep-learning network. Ecological Informatics 83:102794. https://doi.org/10.1016/j.ecoinf.2024.102794

Vilar-Andreu M, García L, Garcia-Sanchez AJ, et al (2024) Enhancing precision agriculture pest control: A generalized deep learning approach with yolov8-based insect detection. IEEE Access 12:84420–84434. https://doi.org/10.1109/ACCESS.2024.3413979

Wang D, Wang Y, Li M, et al (2021) Using an improved yolov4 deep learning network for accurate detection of whitefly and thrips on sticky trap images. Transactions of the ASABE 64(3):919–927. https://doi.org/10.13031/trans.14394

Wang F, Liu L, Dong S, et al (2022) Asp-det: Toward appearance-similar light-trap agricultural pest detection and recognition. Frontiers in Plant Science 13:864045. https://doi.org/10.3389/fpls.2022.864045

Wang N, Fu S, Rao Q, et al (2025) Insect-yolo: A new method of crop insect detection. Computers and Electronics in Agriculture 232:110085. https://doi.org/10.1016/j.compag.2025.110085

Wang QJ, Zhang SY, Dong SF, et al (2020) Pest24: A large-scale very small object data set of agricultural pests for multi-target detection. Computers and Electronics in Agriculture 175:105585. https://doi.org/10.1016/j.compag.2020.105585

Wang S, Chen D, Xiang J, et al (2024) A deep-learning-based detection method for small target tomato pests in insect traps. Agronomy 14(12). https://doi.org/10.3390/agronomy14122887

Wei M, Zhan W (2024) Yolo_mrc: A fast and lightweight model for real-time detection and individual counting of tephritidae pests. Ecological Informatics 79:102445. https://doi.org/10.1016/j.ecoinf.2023.102445

Xiang Q, Huang X, Huang Z, et al (2023) Yolo-pest: An insect pest object detection algorithm via cac3 module. Sensors 23(6). https://doi.org/10.3390/s23063221

Yao Q, Feng J, Tang J, et al (2020) Development of an automatic monitoring system for rice light-trap pests based on machine vision. Journal of Integrative Agriculture 19(10):2500–2513. https://doi.org/10.1016/S2095-3119(20)63168-9

Zhang W, Huang H, Sun Y, et al (2022) Agripest-yolo: A rapid light-trap agricultural pest detection method based on deep learning. Frontiers in Plant Science 13:1079384. https://doi.org/10.3389/fpls.2022.1079384

Zhang W, Wang Y, Shen G, et al (2025) Detection of bemisia tabaci based on swinir super-resolution reconstruction and semantic-sam model. Computers and Electronics in Agriculture 237:110667. https://doi.org/10.1016/j.compag.2025.110667

Zhang Z, Rong J, Qi Z, et al (2024) A multi-species pest recognition and counting method based on a density map in the greenhouse. Computers and Electronics in Agriculture 217:108554. https://doi.org/10.1016/j.compag.2023.108554

Zhou C, Lee WS, Zhang S, et al (2024) A smartphone application for site-specific pest management based on deep learning and spatial interpolation. Computers and Electronics in Agriculture 218:108726. https://doi.org/10.1016/j.compag.2024.108726
